# Mid-upper arm circumference (MUAC) measurement usage among children with disabilities: A systematic review

**DOI:** 10.1177/02601060231181607

**Published:** 2023-06-20

**Authors:** Julia Hayes, Michael Quiring, Marko Kerac, Tracey Smythe, Cally J Tann, Nora Groce, Zerihun Gultie, Lydia Nyesigomwe, Emily DeLacey

**Affiliations:** 1 Nutrition and Health Services, Holt International, Eugene, Oregon, USA; 2 Department of Population Health, 170945 Faculty of Epidemiology and Population Health, London School of Hygiene & Tropical Medicine, University of London, London, UK; 3 Centre for Maternal, Adolescent, Reproductive, & Child Health (MARCH), London School of Hygiene & Tropical Medicine, University of London, London, UK; 4 International Centre for Evidence in Disability, Department of Population Health, 170945 Faculty of Epidemiology and Population Health, London School of Hygiene & Tropical Medicine, University of London, London, UK; 5 Division of Physiotherapy, Department of Health and Rehabilitation Sciences, 26697 Stellenbosch University, Cape Town, South Africa; 6 Infectious Disease Epidemiology & International Health, 170945 Faculty of Epidemiology and Population Health, London School of Hygiene and Tropical Medicine, University of London, London, UK; 7 47968 MRC/UVRI & LSHTM Uganda Research Unit, Entebbe, Uganda; 8 Neonatal Medicine, 8964 University College London Hospitals NHS Trust, London, UK; 9 UCL International Disability Research Centre, Department of Epidemiology and Health Care, University College London, London UK; 10 Holt International Ethiopia, Addis Ababa, Ethiopia; 11 Holt International Uganda, Kampala, Uganda

**Keywords:** Disability, Child nutrition, Nutritional status, Undernutrition, Assessment of nutritional status, Mid-upper arm circumference, Anthropometry

## Abstract

**Background:** Anthropometric measurements, including mid-upper arm circumference (MUAC), are used for monitoring and evaluating children's nutritional status. Evidence is limited on optimal nutritional assessment for children with disabilities, who are at high risk for malnutrition. **Aim:** This study describes MUAC use among children with disabilities. **Methods:** Four databases (Embase, Global Health, Medline, and CINHAL) were searched from January 1990 through September 2021 using a predefined search strategy. Of the 305 publications screened, 32 papers were included. Data included children 6 months to 18 years old with disabilities. Data including general study characteristics, methods for MUAC measurement, terminology, and measurement references were extracted into Excel. Due to heterogeneity of the data, a narrative synthesis was used. **Results:** Studies from 24 countries indicate that MUAC is being used as part of nutritional assessment, but MUAC measurement methods, references, and cutoffs were inconsistent. Sixteen (50%) reported MUAC as a mean ± standard deviation (SD), 11 (34%) reported ranges or percentiles, 6 (19%) reported z-scores, and 4 (13%) used other methods. Fourteen (45%) studies included both MUAC and weight-for-height but nonstandard reporting limited comparability of the indicators for identifying those at risk of malnutrition. **Conclusion:** Although its speed, simplicity, and ease of use afford MUAC great potential for assessing children with disabilities, more research is needed to understand its appropriateness, and how it performs at identifying nutritionally high-risk children in comparison to other measures. Without validated inclusive measures to identify malnutrition and monitor growth and health, millions of children could have severe consequences for their development.

## Introduction

The 1989 International Convention on the Rights of the Child recognizes the rights of the child as human rights with consideration for their vulnerability and needs (UN General Assembly, 1959). The global community is in consensus that all children have the right to have their fundamental needs met. Children have the right to good nutrition, care, and support that will ensure their full development (UN General Assembly, 1959, 2015; UNICEF, 2021; United Nations Children's Fund, 2021). Countries and the global community, therefore, are accountable to uphold these children's rights (UN General Assembly, 1959, 2015; UNICEF, 2021; United Nations Children's Fund, 2021). When considering the needs of all children, it is important to recognize those whose rights are marginalized, including many of the nearly 240 million children worldwide living with disabilities (United Nations Children's Fund, 2021). Disability refers to the interaction between physical, mental, or intellectual impairments and a child's environment, which can limit and even restrict their full participation in activities (World Health Organization, 2011).

Childhood is a critical period of growth and development and malnutrition during this time can result in lifelong consequences to health, cognitive and neurobehavioral growth, growth, and limited educational or economic attainment later in life (Gakidou et al., 2017). Malnutrition is defined as “Any condition in which deficiency, excess, or imbalance of energy, protein, or other nutrients… adversely affects body function and/or clinical outcome” (Meier and Stratton, 2008). Millions of children worldwide are impacted by malnutrition, including undernutrition, overnutrition, and micronutrient deficiencies. In 2020, 149 million children were stunted and 45 million were wasted (UNICEF-WHO-World Bank: Joint Child Malnutrition Estimates - 2021 edition interactive dashboard - UNICEF DATA, n.d.). Malnutrition is linked to more than 1 million deaths and 3.8% of the disability-adjusted life years (DALYs) lost globally (Gakidou et al., 2017).

Children with disabilities are at an increased risk of malnutrition (Black et al., 2013a; Black et al., 2013b; DeLacey et al., 2020, 2021, 2022; Groce et al., 2014). Children with disabilities are 25% more likely to be wasted and 34% more likely to be stunted than those without disabilities (United Nations Children's Fund, 2021). This increased risk can be directly and indirectly related to underlying impairments or children's environments. Health and medical conditions that require additional care or present feeding challenges, in addition to economic, social, and cultural norms, can all increase children's risk of malnutrition (United Nations Children's Fund, 2021; DeLacey et al., 2020, 2021, 2022; Groce et al., 2014). Despite being a high-risk population, children with disabilities are often neglected in malnutrition guidelines and determining nutritional status or appropriate assessment methods may be difficult (United Nations Children's Fund, 2021; Engl et al., 2022; Hardy et al., 2018).

Anthropometry is a reliable common way of assessing nutritional status and growth patterns of individuals (World Health Organization Expert Committee, 1995). Common measures include weight, height or length, head circumference, and mid-upper arm circumference (MUAC; Fryar et al., 2021). Anthropometric measures are routinely taken at medical check-ups and plotted on growth charts to illustrate growth patterns and inform clinicians of a child's growth and nutritional status (Fryar et al., 2021). Consecutive anthropometric measurements can help identify abnormal growth patterns, which could be a sign of underlying medical, nutritional, or psychosocial problems (Child Health and Disability Prevention Program, 2016). The World Health Organization's child growth standards and references are commonly used and offer a standardized way to assess anthropometric measures in z-scores or percentiles (World Health Organization, 2007, 2022).

Mid-upper arm circumference is an anthropometric measure that originated in the 1950s (Glasman, 2018). MUAC measurement is widely used because it offers a simple, quick way to identify children at high risk of malnutrition that does not need to be adjusted for age or sex (though MUAC z-score and percentile tables do exist by age and sex) (World Health Organization, 2022). For children 6 months to 5 years of age, the WHO's recommended cutoffs are 11.5 cm for severe malnutrition and between 11.5 cm and 12.5 cm for moderate malnutrition (World Health Organization, 2013). To help with assessment, MUAC tapes are often color coded so that even those with limited numeracy can easily use and interpret them in community settings (Bliss et al., 2018; MUAC,Child 11.5 Red,PAC-50,English, n.d.). Although there currently are not internationally agreed-upon cutoffs for children older than 5 years, research on its application among older children continues, with the potential for MUAC to provide insights into the nutritional status of children of all ages (Shinsugi et al., 2020; World Health Organization, 2009a). However, there is limited information on if MUAC has the potential to be an appropriate or useful measure of malnutrition risk for children with disabilities.

### Aim and objectives

The aim of our review is to describe the use of MUAC measurement among children with disabilities. Our objectives were to:

1. Describe the use of MUAC measurement in the assessment of nutritional status of children with disabilities.
2. Examine the use of MUAC in relation to other anthropometric measurements or the use of MUAC between groups of children (e.g., comparison of those with disabilities to those without disabilities where possible).
3. Explore the usability of current MUAC cutoff values or MUAC z-scores as part of assessing the nutritional status of children with disabilities.

## Methods

### Search strategy

Following PRISMA guidelines, we analyzed existing published peer-reviewed literature on the use of MUAC among children with disabilities (S1) (Page et al., 2021). A PICOS framework was used to develop the research question (Table 1), and a PROSPERO registration was completed prior to the start of the study [PROSPERO 2021 CRD42021258027 Available from: https://www.crd.york.ac.uk/prospero/display_record.php?ID=CRD42021258027](Aslam and Emmanuel, 2010). Ethical approval for this systematic review was determined not to be required by the [University Ethics Reference].

**Table 1.** PICOS criteria for search strategy.

PICOS criteria	
Population	Children between the ages of 6 months and 18 years with one or more disabilities
Intervention	Use of MUAC measurement
Comparator	Any study type including observational studies
Outcomes	Description of use of MUAC and any other anthropometric measurements (WHZ, WAZ, HAZ, BMIZ, HCAZ) and related nutritional information.
Setting	Any country or geographical region

### Inclusion/exclusion criteria

Inclusion criteria included studies published in English from January 1990 through September 2021, which contained research on children with disabilities and MUAC measurements (Table A1). Studies needed to include at least one measurement of MUAC. Other anthropometric indicators were included for comparison (e.g., length/height-for-age, weight-for-age, weight-for-length/height, and BMI-for-age) where available. Full-text cross-sectional studies, case–control studies, cohort studies, and randomized controlled trials conducted in all geographic areas were eligible for inclusion. Studies were excluded if they were conducted in intensive care settings or if MUAC values were used to calculate other arm measurements including upper arm muscle area (UAMA) and upper arm fat area (UAFA) but no values for MUAC were included.

### Study selection

JH, ED, and MQ determined and tested the appropriate search strategy. The search strategy was developed with guidance from a search strategy from Banks et al. (Table A2) (Banks et al., 2017). JH and ED applied the finalized search strategy from September 8, 2021, through September 29, 2021. Two electronic databases were searched through OVID, Embase, and Global Health, and two electronic databases were searched through EBSCO Host, PubMed/Medline, and CINHAL Plus. JH and ED independently completed initial title and abstract screening of articles identified by the search strategy. One additional paper was identified for inclusion through other methods. Papers identified by JH and ED as eligible for possible inclusion and full-text review were then analyzed by JH, MQ, and ED against the predetermined inclusion/exclusion criteria. Any discords in the inclusion of full-text studies were discussed among JH, MQ, and ED with ED deciding any discords.

### Data extraction and analysis

Studies included were imported into EndNote X9 v12 and Mendeley Desktop v1.19.8 for review, synthesis, and coding (The EndNote Team, 2013; The Mendeley Team, 2008). We undertook data extraction using a standardized form that included study design, location, population, age range, sex representation, disability type, and setting (Table 2a and Table 2b). Data were extracted on methods for MUAC measurement with any variations in terminology, measurement references, or measurement techniques as reported. Z-scores and percentiles for MUAC and other anthropometric measures were included where available.

**Table 2a.** Characteristics of studies included in the full text analysis.

Characteristic	*N* (%)
Study design	*N* = 32 studies
Cross-sectional	18 (56%)
Case–control	5 (16%)
Cohort	8 (25%)
Randomized control trial	1 (3%)
Years of publication	
1990–1999	2 (6%)
2000–2010	12 (38%)
2011–2019	11 (34%)
2020–2022	7 (22%)
WHO region/country	
African Region	5 (16%)
Region of the Americas	7 (22%)
South-East Asia Region	6 (19%)
European Region	6 (19%)
Eastern Mediterranean Region	5 (16%)
Western Pacific Region	3 (9%)
Multiregion	1 (3%)
Country Classification	
Low income	4 (13%)
Lower-middle income	16 (50%)
Upper-middle income	9 (28%)
High income	8 (25%)
Disability types appearing in multiple studies	
Cerebral palsy	14 (44%)
Intellectual impairment	6 (19%)
Visual impairment	3 (9%)
Autism spectrum disorder	3 (9%)
Sickle cell disease	2 (6%)
Down syndrome	2 (6%)
Epilepsy	2 (6%)
Hearing impairment	2 (6%)

**Table 2b.** Description of studies included in the review of use of mid-upper arm circumference (MUAC) among children with disabilities.

Author/year	Study design	Country	Sample size	Age range	% Female	Disability type(s)	Setting
Dannhauser et al., 2007	Cross-sectional	South Africa	*N* = 145	8–15 years	Not available	Multiple (mental disability, physical disability, and/or learning disability)	School
Kakooza-Mwesige et al., 2015	Cohort	Uganda	*N* = 135	2–12 years	47%	Cerebral palsy	Clinic and hospital
Kuper et al., 2015	Case–control	Kenya	*n* = 311 *n*^†^ = 496	6 months to 10 years	38%	Multiple (physical impairment, epilepsy, visual impairment, hearing impairment, and intellectual impairment)	County
Lelijveld et al., 2016	Cohort	Malawi	*n* = 352 *n*^†^ = 401	7.4–12.8 years	Not available	Multiple (“clinically obvious disability” unspecified), HIV, Noncommunicable diseases	City
Tompsett et al., 1999	Cross-sectional	Nigeria	*n* = 112 *n*^†^ = 199	<10 years	44%	Multiple (poliomyelitis, neurological, orthopedic, learning difficulties and/or sensory impairments)	Multiple regions
Barnhill et al., 2017	Case–control	USA Region of the Americas	*n* = 86 *n*^†^ = 57	2–13 years	12%	Autism spectrum disorder	Unspecified
Bartlett et al., 2010	Cohort	Canada	*N* = 135	11.6–17.9 years	44%	Cerebral palsy	City
Caminiti et al., 2018	Case–control	Argentina	*N* = 131	0.7–18.6 years	50%	Myelomeningocele	Hospital
Kuperminc et al., 2010	Cohort	USA	*N* = 58	8–18 years	43%	Cerebral palsy	Unspecified
Saldanha Tschinkel et al., 2018	Cross-sectional	Brazil	*N* = 23	1–12 years	57%	Autism spectrum disorder	City
Silva et al., 2017	Cross-sectional	Brazil	*N* = 68	2–11 years	31%	Cerebral palsy	Hospital
Zemel et al., 2002	Randomized control trial	USA	*N* = 42	4–10 years	48%	Sickle cell disease	Hospital
Freeman et al., 2002	Case–control	India	*n* = 41 *n*^†^ = 40	2–7 years	Not available	Multiple neurological disabilities (mainly cerebral palsy)	Urban community
Hussain et al., 1996	Cross-sectional	Bangladesh	*n* = 105 *n*^†^ = 105	2–15 years	Not available	Night blindness	Rural community
Jahan et al., 2021c	Cross-sectional	Indonesia	*N* = 130	<18 years	44%	Cerebral palsy	Rural community
Jahan et al., 2021a	Cross-sectional	Nepal	*N* = 182	5.3–15.3 years	74%	Cerebral palsy	Rural community
Pai et al., 2001	Case–control	India	*N* = 129	2–10 years	52%	Multiple (motor impairments, neurological impairments, speech impairments, learning impairments and/or epilepsy)	School
Rose-Clarke et al., 2019	Cross-sectional	India	*N*^†^ = 3324 *n* = 14	10–<19 years	100%	Unspecified	Rural community
Leonard et al., 2020	Retrospective cohort	Belgium	*N* = 260	18 months to 18 years	43%	Cerebral palsy	Hospital
Sahin and Nogay, 2021	Cross-sectional	Turkey	*N* = 122	4–18 years	44%	Intellectual disabilities	Rehab centers
Samara et al., 2010	Cohort	UK/Ireland	*N* = 223	6 years	44%	Multiple (eating problems, behavioral disabilities, and extremely preterm)	Unspecified
Soylu et al., 2008	Prospective Interventional Cohort	Turkey	*n* = 45	1.9–9.1 years	36%	Spastic quadriplegia	Hospital
Tekin et al., 2018	Cross-sectional	Turkey	*N* = 1057	1.8–12.6 years	43%	Multiple (epilepsy, cerebral palsy, neuromuscular disorders, neurometabolic disorder, neuroimmune disorders)	Clinic
Troughton and Hill, 2001	Cross-sectional	Ireland	*N* = 93	2.6–18.7 years	38%	Cerebral palsy	School
Al-Saqladi et al., 2010	Cross-sectional	Yemen	*N* = 102	6 months to 15 years	45%	Sickle cell disease	Hospital
Hamza et al., 2015	Cross-sectional	Egypt	*N* = 84	6 months to 15.5 years	55%	Osteogenesis imperfecta	Clinic and hospital
Kotby et al., 2020	Cross-sectional	Egypt	*N* = 80	7 months to 4.75 years	40%	Down syndrome	Hospital
Saleem et al., 2021	Cross-sectional	Pakistan	*N* = 200	6 months to 4.9 years	50%	Multiple (delayed social development, fine motor development, gross motor development, and global development)	Clinic and rural health center
Tomoum et al., 2010	Cross-sectional	Egypt	*n* = 40 *n*^†^ = 40	2–8 years	48%	Cerebral palsy	Hospital
Ahmad et al., 2020	Cross-sectional	Malaysia	*N* = 93	5–17 years	45%	Cerebral palsy	Community-based rehabilitation centers
Zainah et al., 2001	Cross-sectional	Malaysia	*n* = 101 *n*^†^ = 101	2–12 years	41%	Cerebral palsy	Clinic, hospital, and community-based rehabilitation centers
DeLacey et al., 2021	Retrospective cohort	China, India, Mongolia, the Philippines, Ethiopia, Vietnam	*N*^†^ = 2926 *n* = 739	0–18 years 0–6 months: 746 6–12 months: 245 12–24 months: 282 24–59 months: 427 (14.6%) 5–18 years: 1226 (41.9%)	49%	Multiple (autism spectrum disorder, cerebral palsy, cleft lip/cleft palate, cognitive impairment, Down syndrome, hearing loss/deafness, heart disease/defect, HIV/AIDS, hydrocephaly, microcephaly, vision impairment and blindness, speech/language delays, others)	IBC

*Note:* n: children with disabilities, n^†^: children without disabilities, N: total population of only children with disabilities, N^†^: total population mixed of children with and without disabilities, IBC: Institution-based care.

Children with specific disabilities were extracted for subgroup analysis if there was sufficient data (e.g., use of MUAC among children with cerebral palsy). Heterogeneity in the data available limited our ability to conduct a meta-analysis, so a narrative synthesis was used.

### Critical appraisal

The JBI Critical Appraisal Tool for appraisal of cross-sectional studies, cohort studies, case–control studies, and randomized control trials was used to assess the papers (Moola et al., 2020). Critical appraisal of each study using the appropriate tool for study type can be found in Table A3.1 to Table A3.4.

## Results

### Study selection

**Figure 1.**
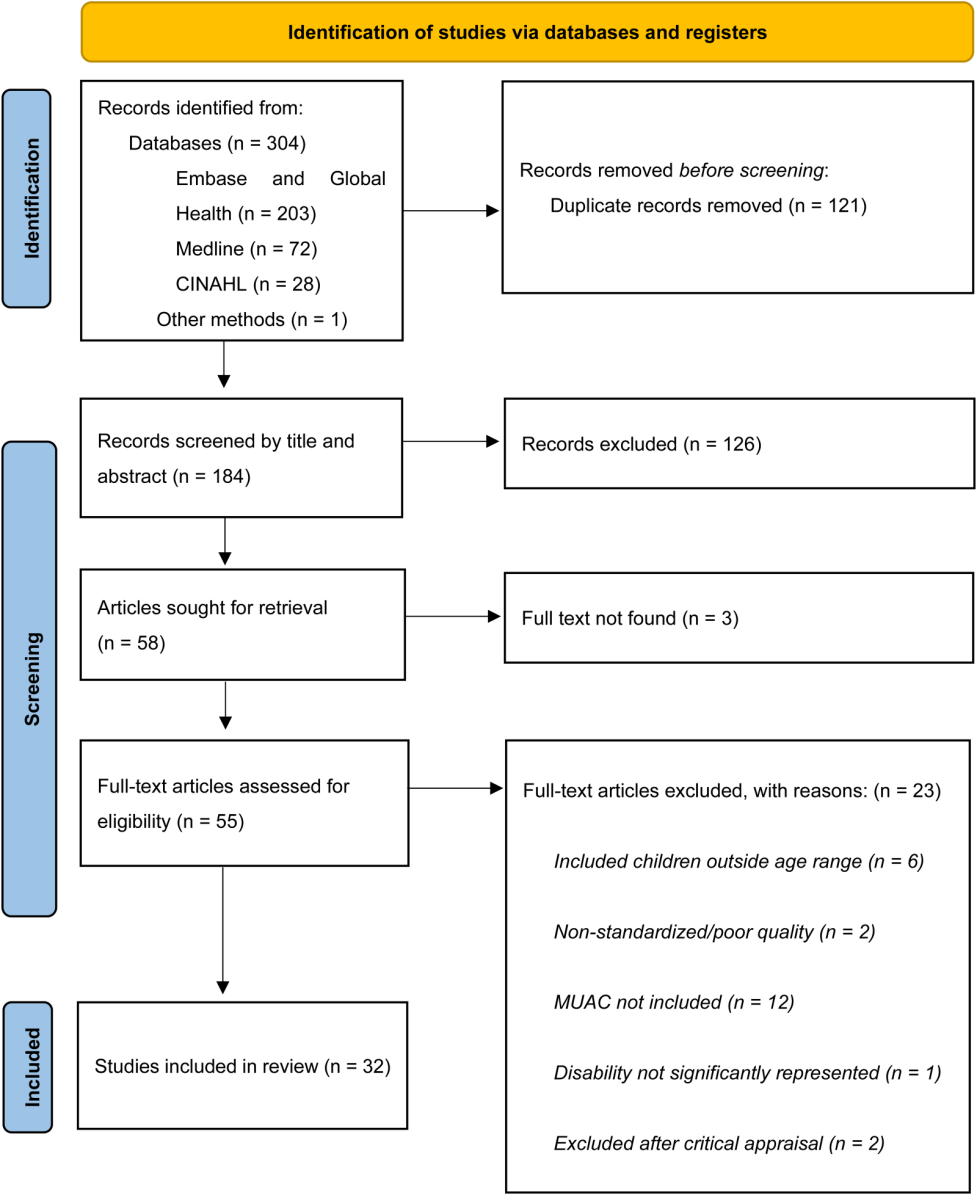
PRISMA flow diagram.

The database search generated 304 studies and one study was found through other methods. After 121 duplicates were removed, 184 records were screened by title/abstract using the inclusion and exclusion criteria, of which 58 were eligible for full-text review (Figure 1). Following a full-text review and critical appraisal, 32 studies were determined to meet the inclusion criteria and 23 studies were excluded (Figure 1, Table A3 and Table A5).

### Study characteristics

Most of the studies included in the review were observational studies (29/32, 91%), representing 26 different countries. India, Egypt, and the United States were each represented in 3/32 (9%) of the studies. Of the included studies, over half (17/32, 53%) were published in the past 5 years (2017 through 2021) (Table 2b). The median age of children in the research was 8 years. The most common types of disability reported were cerebral palsy, intellectual impairment, and autism spectrum disorder (Table 2a). Nine studies (29%) included more than one type of disability. Gender was reported in 28 studies (88%). The average female representation among 28 studies was 47%.

There was a wide geographic distribution based on WHO regions. The most common was the Americas represented in seven studies (22%). The European Region and South-East Asia Region were represented in six studies (19%), and the African Region and the Eastern Mediterranean Region both represented in five studies (16%) (Table 2a, Table A4). The distribution of studies among countries of different income levels was also widespread. The most common level represented was lower-middle income countries in 16 studies (50%). Upper-middle income followed, represented in nine studies (28%) followed by high income represented in eight studies (25%).

### Anthropometric indicators

Varying anthropometric measurements were reported including weight-for-length/height, length/height-for-age, weight-for-age, BMI/BMI-for-age, weight, height, head circumference, waist circumference, triceps skinfold thickness, and subscapular skinfold thickness (Table 3). Besides MUAC, the most common anthropometric indicator was height-for-age found in 25 (78%) of studies, followed by weight-for-age (24/32, 75%), BMI (20/32, 63%) and weight-for-length/height (14/32, 44%). Additional indicators, such as head circumference-for-age, were reported in several of the studies (20 (63%)).

**Table 3.** Mid-upper arm circumference (MUAC) measurement, presentation, and growth references used for other anthropometry, cutoffs, and methods of measurement.

Author, year	Type of disability	References used for presentation of anthropometry	Cutoffs if specified	Method of MUAC Measurement					
				Midpoint of arm	Left arm	90° angle of arm	MUAC tape	Nearest 0.1 cm	Additional information on method
Hussain et al., 1996	Night blindness	MUAC: US NHANES (Jeliffe and Jeliffe, 1989) WFH: National Center for Health Statistics reference data (Hamill et al., 1977)	MUAC Normal: ≥85% Moderate 80–84% Severe: < 80% WFH Severely wasted: ≤−3 standard deviations Moderately wasted: −2 to −3 standard deviations		X				Upper arm measured to the nearest mm Oil-cloth tailor's tape
Tompsett et al., 1999	Multiple (poliomyelitis, neurological, orthopedic, learning difficulties and/or sensory impairments)	National Center for Health Statistics reference data for WHZ and other anthropometry. Unadjusted simple MUAC presented.			X			X	
Zainah et al., 2001	Cerebral palsy	MUAC: Frisancho, 1981 WFH: Hamill et al., 1979			X				Upper arm Nonstretch measuring tape
Troughton and Hill, 2001	Cerebral palsy	Frisancho, 1981	MUAC Undernutrition: < 5th centile						Harpenden plastic tape Average of at least 2 measurements
Pai et al., 2001	Multiple (motor impairments, neurological impairments, speech impairments, learning impairments and/or epilepsy)	MUAC: Trowbridge, 1979 WFH: National Center for Health Statistics reference data	WFH Z-score Moderate-severely wasted: < −2 standard deviations MUAC Moderate and severe malnutrition: < 13.5 cm					X	
Freeman et al., 2002	Multiple neurological disabilities (mainly cerebral palsy)	National Center for Health Statistics reference data for WHZ and other anthropometry. Unadjusted simple MUAC presented.							Unspecified
Zemel et al., 2002	Sickle cell disease	National Center for Health Statistics reference data						X	Nonstretchable tape Average of 3 measurements
Dannhauser et al., 2007	Multiple (mental disability, physical disability and/or learning disability)	National Center for Health Statistics reference data							Right side Average of 3 measurements
Soylu et al., 2008	Cerebral palsy	National Center for Health Statistics reference data for WFH Unadjusted MUAC presented		X	X				Arm hanging down Nonstretching tape
Tomoum et al., 2010	Cerebral palsy	Krick et al., 1996							Nonstretchable stainless-steel tape Average of 3 measurements
Al-Saqladi et al., 2010	Sickle cell disease	WHO Growth Standards for WFH and MUAC for Age [6months to 5 years] (World Health Organization, 2022) WHO Reference Data for weight for height of older children (World Health Organization, 2007)	WFH z-scores Moderate wasting: ≤−2 standard deviations Severe wasting: ≤−3 standard deviations	X	X			X	Arm hanging loosely Nonextensible fiberglass tape
Bartlett et al., 2010	Cerebral palsy	Unspecified			X				Left side of body Measured in cm
Kuperminc et al., 2010	Cerebral palsy	Frisancho, 1990							Average of 2 measurements
Samara et al., 2010	Multiple (eating problems, behavioral disabilities, and extremely preterm)	Unspecified							Average of 2 measurements LASSO-O tape
Kakooza-Mwesige et al., 2015	Cerebral palsy	WHO Growth Standards						X	Tape measurer Average of 2 measurements
Kuper et al., 2015	Multiple (physical impairment, epilepsy, visual impairment, hearing impairment, and intellectual impairment)	WHO Growth Standards [MUAC for Age and other anthropometry]	MUAC Low MUAC: ≤−2 standard deviations WFH Wasted: ≤−2 standard deviations					X	Child tapes Average of 3 measurements
Hamza et al., 2015	Osteogenesis imperfecta	Frisancho, 1990	WFH Underweight: < –2 standard deviations	X	X	X		X	Average of 3 measurements Conventional nonstretchable tape
Lelijveld et al., 2016	Multiple (“clinically obvious disability” unspecified), HIV, Noncommunicable diseases	WHO Growth Reference Data							Measured in mm
Silva et al., 2017	Cerebral palsy	WHO Growth Standards							Inextensible tape Three measurements obtained, closest two were averaged and reported
Barnhill et al., 2017	Autism spectrum disorder	Unspecified							Unspecified
Caminiti et al., 2018	Myelomeningocele	≤60 months: WHO Growth Standards >60 months: National Committee for Growth and Development Argentine Society of Pediatrics, 2013 (Comité Nacional de Crecimiento y Desarrollo, 2013)		X					Nonextensible tape measure Measured in cm
Saldanha Tschinkel et al., 2018	Autism spectrum disorder	WHO Growth Standards		X		X			Anthropometric tape fixed on marked point
Tekin et al., 2018	Multiple (epilepsy, cerebral palsy, neuromuscular disorders, neurometabolic disorder, neuroimmune disorders)	National Center for Health Statistics reference data	WFH Normal: > 90% Mild malnutrition: 80–90% Moderate malnutrition: 70–0% Severe malnutrition: < 70%	X	X				Arm flexed slightly at elbow Plastic measuring tape
Rose-Clarke et al., 2019	Unspecified	WHO Growth Reference Data For MUAC reference, see cutoffs.	For adolescent thinness: <160 mm among girls aged 10–14 years based on nutrition guidelines for children with HIV						Standard adult tape (UNICEF) Average of two measurements
Ahmad et al., 2020	Cerebral palsy	World Health Organization. Guidelines for an Integrated Approach to Nutritional care of HIV-infected Children (6 months to 14 years), Geneva: World Health Organization; 2009 (World Health Organization, 2009b)		X					Measured in cm Wrapped around without compression of soft tissue
Leonard et al., 2020	Cerebral palsy	MUAC: Fryar et al., 2012 WFH: Life Expectancy, 2011	WFH Acute malnutrition: < 90% Moderate malnutrition: 80–89% Severe malnutrition: < 80%						Unspecified
Kotby et al., 2020	Down syndrome	WHO Growth Standards	WFH Z-scores Overweight: > 2 standard deviations Normal: −2 to –2 standard deviations Wasted: < −2 standard deviations		X				Upright with arm down in a fully relaxed position Tape measure perpendicular to the long axis of the arm No pinching or gaping of the tape
Saleem et al., 2021	Multiple (delayed social development, fine motor development, gross motor development, and global development)	WHO Growth Standards	MUAC Severe malnutrition: < 115 mm WFH Z-scores Severe wasting: < −3 standard deviations						Unspecified
Jahan et al., 2021a	Cerebral palsy	WHO Growth Reference Data	MUAC and WFH Z-scores Overnutrition:>+2 standard deviations Normal: −2 to +2 standard deviations Moderately wasted: −2 to −3 standard deviations Severely wasted: ≤−3 standard deviations				X		Measured in cm
Jahan et al., 2021c	Cerebral palsy	WHO Growth Reference Data	MUAC and WFH Z-scores				X		Measured in cm
Sahin and Nogay, 2021	Intellectual disabilities	WHO Growth Reference Data							Appropriate methods (WHO Technical Report Series, 1995)
DeLacey et al., 2021	Multiple (autism spectrum disorder, cerebral palsy, cleft lip/cleft palate, cognitive impairment, Down syndrome, hearing loss/deafness, heart disease/defect, HIV/AIDS, hydrocephaly, microcephaly, vision impairment and blindness, speech/language delays, others)	WHO Growth Reference Data							

*Note:* WHO: World Health Organization; WFH: Weight-for-Length/Height; WHZ: Weight-for-Length/Height z score; WAZ: Weight-for-Age z score; HAZ: Length/Height-for-Age z score; cm: centimeter; mm: millimeter.

### Use of MUAC

The terminology of MUAC varied in the 32 included studies (Table 3). Additional information on MUAC terminology, methods for MUAC measurement, measurement references (e.g., WHO, CDC) and data reported for MUAC and other anthropometric indicators (e.g., weight-for-age, height-for-age, weight-for-length/height, and BMI) are included in Table A4. Although most studies referred to MUAC using MUAC or a variation of all those words, 11 used other variations that did not include MUAC (Table 3). Few studies reported methods for measuring MUAC. Twelve of the studies (12/32, 38%) specified if the measurement was completed on the mid-upper arm, if the left arm was used, and if the arm was at a 90° angle for the measurement and seven (7/32, 22%) noted measurements were obtained to the nearest 0.1 centimeter. Only one study mentioned using a specialized MUAC tape. Nonstandard, limited descriptions of the methods used for MUAC measurement were common among the few studies that reported methods. How MUAC measurements were presented also varied greatly among the studies. Sixteen (50%) studies presented MUAC values as means with standard deviations, 11 (34%) presented MUAC values as the number or percentage of children within a specific percentile, and 6 (19%) used z-scores to present MUAC values obtained for specific groups. Four studies (13%) presented MUAC results in other ways and two studies (6%) did not include MUAC values but did mention MUAC in their methods sections. When grouped by disability type, using means with standard deviations was most common for studies that included children with multiple types of disabilities (6/9, 66%). Several of the studies included children over 5 years in MUAC analysis or did not specify the ages included.

### Reference values for MUAC

MUAC reference values that were used are shown in Table 3. Publication years ranged from 1996 to 2021. Growth references evolved through this period and, therefore, variation in references used for anthropometric measurements is notable. In studies dated 1996 through 2010, the US National Center for Health Statistics (NCHS) data were used most, in six of 14 studies published during this time. In the studies dated 2015 through 2021, WHO growth standards became common, referred to in 16 out of 18 studies published during this time frame. Additionally, three studies noted the use of findings by Frisancho based on NCHS data and the Nutritional Examination Survey of 1971 to 1974 for the assessment of anthropometric measurements (Table 3) (Frisancho, 1981, 1990).

### MUAC vs. Weight-for-length/height

Of the studies included, 15 (47%) studies included measurements for both MUAC and weight-for-length/height (Table 3 and Table 4). Of these studies, four (27%) reported MUAC and weight-for-length/height as means with standard deviations, five (20%) reported each as the number and/or percentage of children with measurements within a specified range or percentile, three (20%) studies reported both as mean z-scores. Among the 12 studies that reported both MUAC and weight-for-length/height with the same method (mean ± standard deviation, number and/or percentage of children with measurements with/in a specified range or percentile, or z-scores), five reported anthropometric data in multiple ways. Four studies did not report MUAC and weight-for-length/height in comparable ways. A comparison of MUAC measurements with weight-for-length/height measurements among these studies is included in Table 4. Of the studies that included measurements for both MUAC and weight-for-length/height only eight reported both with the same methods. Despite the limited comparability of these two indicators in the data, narrative data from the studies suggest that MUAC was a useful measure, especially for children with disabilities for which obtaining height is a challenge.

**Table 4.** Comparison of mid-upper arm circumference and weight-for-length/height anthropometry with age ranges as specified in each study of children included in the entire study or for only those who received MUAC measurements, and/or those who received weight-for-length/height measurements as available.

Author, year	Age range (entire study, MUAC, WFH)	MUAC-based nutritional status	Weight-for-length/height (WFH) -based nutritional status
Studies representing MUAC and WFH results as the number or percentage of children within specified category.			
Hussain et al., 1996	Entire study: 2–15 years	Percent of children within percentile range Normal (≥85%): 11% (8/71) Moderate malnutrition (80–84%): 28% (20/71) Severe malnutrition (<80%): 61% (43/71)	Percent of children with WFH values within specified standard deviations Normal (>−2): 73% (52/71) Moderately wasted:( −2SD to −3SD) 25% (18/71) Severely wasted (←3SD): 2% (1/71)
Jahan et al., 2021c	Entire study: < 18 years MUAC: < 5 years WFH: < 5 years	Percent of children within category Normal: 75% Undernutrition: 8% Severe undernutrition: 17%	Percent of children within category Normal: 75% Undernutrition: 3% Severe undernutrition: 22%
Studies representing MUAC and WFH results as mean with standard deviations (SD) only.			
Soylu et al., 2008	Entire study: 1.9–9.1 years	Mean MUAC in cm ± SD 45 children with disabilities: 14.4 cm ± 2.1 31 children with disabilities: Before Therapy: 14.5 cm ± 2.2 After Therapy: 15.2 cm ± 2.2	Mean WFH percentile ± SD 45 children with disabilities: 89.5% ± 8.3 31 children with disabilities: Before Therapy: 84.1% ± 13.9 After Therapy: 88.7% ± 13.4
Tekin et al., 2018	Entire study: Mean age: 7.2 ± 5.4 years	Mean MUAC in cm ± SD Children with disabilities and malnourished: Baseline: 15.8 cm ± 2.7 6 months: 16.4 cm ± 2.9 Children with disabilities, nonmalnourished: 18.9 cm ± 3.4	Mean WFH percentile ± SD Children with disabilities and malnourished: Baseline: 79.2% ± 9.23 6 months: 81.4% ± 8.17 Children with disabilities, nonmalnourished: 106.4% ± 16.71
Studies representing MUAC and WFH results as z-scores with standard deviations (SD) only.			
		Mean MUAC-for-age z-score ± SD	Mean WFH z-score (WHZ) ± SD
Al-Saqladi et al., 2010	WFH: 6 months to 5 years MUAC: 6 months to 5 years	All: −2.23 ±** **1.02 Male: −2.11 ±** **1.01 Female: −2.29 ±** **1.03 By age: 6–11 months: −2.58 ±** **1.42 12–23 months: −1.67 ±** **0.79 24–35 months: −2.12 ±** **1.07 36–47 months: −2.39 ±** **0.54 48–60 months: −2.34 ±** **1.01	All: −1.38 ±** **1.29 Male: −1.31 ±** **1.24 Female: −1.42 ±** **1.33 By age: 6–11 months: −1.58 ±** **1.48 12–23 months: −0.53 ±** **1.47 24–35 months: −1.17 ±** **1.59 36–47 months: −1.68 ±** **0.85 48–60 months: −1.73 ±** **0.85
Kakooza-Mwesige et al., 2015	MUAC and WHZ: 2–5 years	Mean MUAC-for-age z-score ± SD Children with disabilities: −0.38 ± 1.17	Mean weight-for-height z-score ± SD Children with disabilities: −0.84 ± 1.41
DeLacey et al., 2021	Entire study: 0–18 years MUAC: 6 months–5 years WHZ: 0–5 years	All children: Mean MUAC-for-age z-score: −0.33 ± 1.20 Children with disabilities: mean z-score ± SD 6–12 months: −0.35 (1.58) 12–24 months: −0.70 (1.74) 24–59 months: −0.73 (1.18) Children without disabilities: mean z-score (SD) 6–12 months: −0.20 ± 1.19 12–24 months: −0.16 ± 1.21 24–59 months: −0.37 ± 1.14	All children: Mean weight-for-height z-score: −0.4 ± 1.49 Children with disabilities: mean z-score ± SD 0–6 months: −0.66 ± 1.61 6–12 months: −1.35 ± 1.72 12–24 months: −1.32 ± 1.33 24–59 months: −1.26 ± 1.58 Children without disabilities: mean z-score (SD) 0–6 months: −0.20 ± 1.51 6–12 months: −0.48 ± 1.34 12–24 months: −0.03 ± 1.24 24–59 months: −0.25 ± 1.19
Studies representing MUAC and WFH using different or multiple methods.			
Tompsett et al., 1999	Entire study: younger than 10 years	Mean MUAC in cm ± SD Children with disabilities: 16.0 cm ± 1.6 Sibling control: 15.8 cm ± 1.6 Neighbor control: 15.6 cm ± 1.3	Mean WFH z-score (WHZ) ± SD Children with disabilities: −0.0 ± 1.9 Sibling control: 0.5 ± 2.4 Neighbor control: 0.0 ± 1.9
Pai et al., 2001	Entire study: 2–10 years	Mean MUAC in cm ± SD Children with disabilities: 12.8 cm ± 1.6 Siblings: 13.2 cm ± 1.4 Neighbor Control: 13.0 cm ± 1.3	Mean WFH z-score (WHZ) ± SD Disabled: –1.20 ± 1.26 Siblings: 1.46 ± 1.30 Neighbor Control: 1.05 ± 0.84
Freeman et al., 2002	Entire study: 2–7 years	Mean MUAC in cm ± SD Children with disabilities: Male: 14.72 cm ± 1.27, Female: 15.24 cm ± 1.50 Controls: Male: 15.17 ± 1.38, Female: 14.95 ± 1.17	Mean WHZ ± SD Children with disabilities: Male: −1.23 ± 0.75, Female: −0.98 ± 0.91 Controls: Male: −2.69 ± 0.84, Female: −1.22 ± 0.81
Dannhauser et al., 2007	Entire study: 8–15 years	Mean MUAC in cm ± SD Location 1: 19.2 cm ± 6.2 Location 2: 17.2 cm ± 4.9 Location 3: 17.0 cm ± 4.4 Number (percentage) of children in percentile category—results listed in order of location 1, location 2, location 3 (<5): 2 (15.4%), 2 (3.2%), 0 (0%) (5 to <15): 2 (15.4%), 26 (41.2%), 38 (56.7%) (15 to < 85): 7 (53.8%), 32 (50.8%), 26 (38.8%) (85 to < 95): 1 (7.7%), 1 (1.6%), 1 (1.5%) (≥95): 1 (7.7%), 2 (3.2%), 2 (3.0%)	Number (percentage) of children within z-score deviation category—results listed in order of location 1, location 2, location 3 (< –3): 0 (0%), 0 (0%), 3 (4.5%) (–3 to < –2): 0 (0%), 1 (1.6%), 1 (1.5%) (–2 to < –1): 1 (7.7%), 2 (3.1%), 6 (9.0%) (–1 to < 1): 1 (7.7%), 12 (18.8%), 13 (19.4%) (> 1 to < 2): 0 (0%), 2 (3.1%), 3 (4.5%) (≥ 2): 11 (84.6%), 47 (73.4%), 41 (61.1%)
Kuper et al., 2015	Entire study: 6 months to 12 years	Number (percentage) of children in percentile category Low MUAC for age (z-score ≤ −2): Children with disabilities: 39/155 (25%) Sibling control: 17/113 (15%) Neighborhood control: 17/165 (10%)	Number (percentage) of children in z-score deviation category Low WFH (z-score ≤ −2): Children with disabilities: 39/120 (33%) Sibling control: 26/112 (23%) Neighborhood control: 31/153 (20%) Mean WHZ ± SD Children with disabilities: −1.5 ± 1.4
Kotby et al., 2020	Entire study: 7 months to 4.75 years	Mean MUAC in cm ± SD Overall: 13.65 cm ± 2.46 Children with disabilities: 13.79 cm ± 2.79 Controls: 15.41 cm ± 2.29	Number (percentage) of children in z-score deviation category Wasted (←2): 9/80 (11.3%) Normal (−2 to 2): 55/80 (68.8%) Overweight (>2): 16/80 (20%)
Saleem et al., 2021	Entire study: 6 months to 59 months	Mean MUAC in cm ± SD Children with disabilities: 10 cm ± 0.98 Controls: 14 cm ± 1.19	Mean WHZ ± SD Children with disabilities: −4.07 ± 1.25 Control: 0.40 ± 1.27
Jahan et al., 2021a	Entire study: Mean age: 10.3 ± 5.0 years	Mean MUAC-for-age z-score ± SD Children with disabilities: −0.9 ± 1.4 Number (percentage) of children within z-score deviation category Overnutrition: (z score: >+2 SD): 0/28 (0%) Normal: (z score: −2 SD to + 2 SD): 21/28 (75%) Moderate undernutrition: (z score: > −3 SD to < −2.0 SD): 3/28 (10.7%) Severe undernutrition: (z score: ≤ −3.0 SD): 4/28 (14.3%)	Mean WHZ ± SD Children with disabilities: −0.5 ± 1.6 Number (percentage) of children within z-score deviation category Overnutrition: (z score: >+2 SD): 1/26 (3.8%) Normal: (z score: −2 SD to + 2 SD): 21/26 (80.8%) Moderate undernutrition: (z score: > −3 SD < −2.0 SD): 2/26 (7.7%) Severe undernutrition: (z score: ≤ −3.0 SD): 2/26 (7.7%)

*Note:* SD: standard deviation; WFH: Weight-for-Length/Height; WHZ: Weight-for-Length/Height z score; cm: centimeters.

## Discussion

We found that MUAC is being used in the assessment of the nutritional status of children of all ages with various disabilities in several countries within different settings. However, methods for obtaining MUAC measurements and reporting methods varied markedly. The 32 papers included in this review indicated that most children with disabilities were able to be measured using MUAC as part of assessments examining nutritional status, although few studies compared MUAC to other anthropometry or analyzed the findings within the broader context of changes in nutritional status or long-term health outcomes. There were also limited data on how MUAC use compared for children with disabilities to counterparts without disabilities and only limited reference to the use of MUAC for children with disabilities older than 5 years of age, despite several of the studies including children over 5 years of age in their analysis, and there are no internationally agreed-upon MUAC references for older children. Additionally, none of the studies examined MUAC's ability to identify children at high risk for malnutrition-associated adverse outcomes (notably mortality, morbidity, and impaired neurodevelopment in comparison to other indicators. This limited the comparability of the prevalence and severity of undernutrition as identified by MUAC-based assessment versus weight-for-length/height z-score-based assessment. Standardized references for cutoffs available at the time of publication were mostly used, however, not all studies referenced the guidelines used for MUAC specifically. Together, these are notable findings because the lack of clear standardized reporting limits the examination of the applicability of MUAC for children with disabilities.

### Anthropometry and use of MUAC in nutritional assessment

Anthropometry is key to assessing nutritional status and evaluating children's growth and health (World Health Organization, 2007, 2022). It is not, however, a direct measure of nutrition and there is no single “gold standard” anthropometric measure (Kerac et al. 2020). All have benefits and limitations and are best presented together to evaluate clinically relevant health and developmental risks. MUAC is commonly used in conjunction with other anthropometric measurements as a part of nutritional assessments, in addition to being often used as an independent criterion for identifying children at high risk of mortality and morbidity in resource-limited and humanitarian settings (Myatt et al., 2006; Sørensen et al., 2021). Over the last few decades, the use of MUAC as an anthropometric indicator has evolved, specifically the type of tape used for the measurement and reference cutoffs (Rana et al. 2021). In 1997, the WHO first published a standard reference for MUAC, which was updated in 2007 and then again in 2009 (Glasman, 2018). The widely used threshold for moderate malnutrition (wasting) for children 6 months to 5 years old is under 12.5 cm and under 11.5 cm for severe wasting. These thresholds are also independent admission criterion for most therapeutic and supplementary feeding programs. As part of the new 2006 child WHO growth standards, MUAC-for-age references were also developed (World Health Organization and UNICEF, 2009). MUAC offers some advantages over other measures due to its simplicity, speed, and ease of use, as well as not requiring age and sex adjustment. This makes it suitable for use in most settings including large-scale community services and programs. There is even research that suggests the use of MUAC by families to assess their own children (Blackwell et al., 2015; Bliss et al., 2018).

### Anthropometry for children with disabilities

Older guidelines suggest that anthropometric results from children with disabilities should be disregarded (Tompsett et al., 1999). However, near the turn of the twenty-first century, researchers recognized the importance of resolving the measurement gaps between populations so that all children, regardless of ability, could achieve their right to good nutrition (Child Health and Disability Prevention Program, 2016; Tompsett et al., 1999; UN General Assembly, 1959). Despite this recognition by Tompsett et al. more than 20 years ago, there remains a gap in the research and recommendations for the use of anthropometric measurements among children with disabilities (Child Health and Disability Prevention Program, Health Assessment Guidelines, 2016; Tompsett et al., 1999; United Nations Children's Fund, 2021). The WHO provides clear guidance on anthropometric measurements for children but there is limited or unclear information on the appropriateness of these measures for children with varying types of disabilities (World Health Organization, 2008, 2022). This is also true for other nutritional assessment tools including ESPGHAN, NCHS, and the CDC (Centers for Disease Control and Prevention, 2000; Published Guidelines - ESPGHAN, n.d.).

Several of the studies in this research note the challenges of conducting anthropometric measurements for children with physical disabilities. Disabilities that can present with physical impairments, such as cerebral palsy, were the most common disabilities included in the studies (21/32, 66%). Due to physical characteristics such as body contractures and spinal deformities found in varying degrees of severity among children with cerebral palsy, there are questions about which anthropometric measurements are most appropriate for use in this population (Zainah et al., 2001). Length/height-based measurements for some children with CP and other physical disabilities can be challenging and can either result in missing values or incorrect values—such as underestimating height when a child cannot stand or lie straight. This could result in a falsely high weight-for-height (WFH), missing true cases of malnutrition and misidentifying children eligible and needing to enter nutrition treatment or feeding programs. Troughton and Hill were unable to obtain height measurements in 20% of their study population due to contractures (Troughton and Hill, 2001). Additionally, other disabilities such as Down syndrome and some intellectual disabilities can present with varying composition of body fat and lean masses (González-Agüero et al., 2011; De Lopes et al., 2008). This is important because not being able to get an accurate height measurement limits the application of several other anthropometric indices including weight-for-length/height or BMI-for-age (Hardy et al., 2018). Some research supports the use of other equations or alternative height measurements, such as upper arm length or lower leg length, but these also present with limitations for the use of other standardized references such as BMI-for-age and weight-for-length/height and their associated growth charts (Zainah et al., 2001). Our research found a limited discussion on how varying disabilities may impact MUAC measurement accuracy.

### Use of MUAC for children with disabilities

Our research found that MUAC is commonly being used for children of all ages with disabilities of all types and severities. This highlights the need that health care providers and programs have to track and monitor the growth and nutritional status of children with disabilities—that even without internationally agreed-upon guidance, people are seeking ways to provide inclusive care. However, there was little insight in this research on referral systems to nutrition programs or services for children with disabilities when malnutrition was indicated. Identifying if a child with disabilities falls below the MUAC thresholds, presents the opportunity to have them referred to lifesaving treatment programs and potentially removing another barrier that excludes children with disabilities from services (Bhutta et al., 2017; United Nations Children's Fund, 2021). Our findings suggest that MUAC is a valuable part of multimodal nutritional assessments for children with a wide variety of disabilities, especially when measurements are routinely tracked. Through routine tracking, it becomes possible to gain insights into a child's overall nutritional status and growth patterns (Shinsugi et al., 2020; World Health Organization, 2007, 2009b, 2022). However, the variance in data presentation among the studies reveals that greater research on this topic is necessary for the guidance of the development of accurate and reliable reference data and measurement tools for this population. There were limited and inconsistent descriptions of methods but it is clear that finding methods for identifying children with disabilities who are at high risk for malnutrition is necessary. Not having clear MUAC guidelines for children with disabilities is a serious issue since optimal cutoffs for referral to nutrition support services are unknown and these children may be:

- Smaller than the general population—children with disabilities are more likely to be stunted, wasted, or underweight.
- Have variations in muscle mass and fat deposits related to some genetic disorders or disabilities.
- Larger than the general population— some children with disabilities may mobilize using their arms and thus have a larger-than-normal MUAC.

### Future research

Future research should work toward identifying anthropometric measurements that are appropriate for children with disabilities. For a population with greater needs and at higher risk of nutritional challenges, understanding and supporting their unique growth patterns and development needs is imperative. This research highlights the clear gaps in information on the use of MUAC for children with disabilities. Future research on the use of MUAC should include not only children of all ages but also children with different types of disabilities and present clear standardized methods of MUAC measurement and reference values (United Nations Children's Fund, 2021; Mramba et al., 2017). MUAC presents as a potentially more disability-inclusive measure, especially for those who face challenges in having their length/height measured or have difficulty with being weighed. Clear guidance on its use could enable more children to be reached and included in treatment programs for malnutrition (United Nations Children's Fund, 2021). In combination with other anthropometric measures including weight-for-length/height, length/height-for-age, weight-for-age, and BMI, it could help build a greater foundation of population-specific data to inform future programming, practices, and policies. A key question for examination in future research is whether mortality/morbidity and development risks are the same for children with disabilities with a low MUAC as for children without disabilities with a low MUAC. Do thresholds need to be adjusted to take the disability into account—and if so, which thresholds are for which types of disabilities? WHO guidelines are highly regarded and used to develop malnutrition protocols worldwide, but the lack of disability-specific recommendations leaves children with disabilities underserved. Through future research examining the use of this anthropometric indicator, we can work toward creating more inclusive health systems.

### Strengths and limitations

This study utilized a comprehensive search strategy with inclusive terms for disability. One strength of this research was the studies included came from several countries in different geographical settings of varying income classifications. Of the papers identified, the use of nonstandardized language, measurements, or methods was common. Despite the lack of internationally agreed-upon standards, many of the studies used MUAC for children of all ages and with various disabilities. Additionally, our search strategy included studies from 1990 to 2021 during which research and findings on the use of MUAC have markedly changed and developed. This paper was limited to peer-reviewed research published in English.

Although some research was found, this review did not find enough comparable studies to assess MUAC's ability to identify malnutrition-associated adverse outcomes in children with disabilities compared to those without or compared to other anthropometric indicators or other subgroups of children. This could be related in part to the limited inclusion of children with disabilities in research (DeLacey et al., 2020; United Nations Children's Fund, 2021). Importantly, no study asked the key question of which anthropometric measure best identifies children at high risk of mortality/morbidity/poor development. This information is critical to being able to understand the true benefits of different measures (Mwangome et al., 2012). Neither did any study directly explore the reliability of the different measures as would be ideal to know (Mwangome and Berkley, 2014).

There is the potential for some publication bias within this systematic review, as the focus of these studies often limited what was reported in terms of outcomes and anthropometric indicators. There is a potential risk of measurement bias for the anthropometric measures. Additionally, causes of malnutrition may be underdiagnosed for children with disabilities, possibly related to perceptions of disability in different contexts or for individual children with disabilities who may weigh less or have a reduced height related to their clinical sequelae (Groce et al., 2014; United Nations Children's Fund, 2021). There are also many types of disabilities, but this research was limited by the available data in the studies which included children with various disabilities, who may have been selectively included for their high risk of malnutrition or for other factors such as severity of disability. Furthermore, disabilities are identified in various ways in different countries. Future research in this area could explore utilizing standardized disability identification methods such as the Washington Group Questionnaire (Grellety et al., 2015).

Findings from this study should be used with caution, especially from the studies which included children older than 5 years, for whom there are no internationally agreed-upon MUAC ranges for nutritional assessment. Additionally, the needs of individual children with different types of impairments should be considered; although routine tracking of anthropometric indicators over time should identify children whose growth patterns are flat or declining, interventions to address their growth will vary. Given the biological links between malnutrition, development, and disability, evaluation of the most useful tools to track and monitor children's nutritional status should be prioritized.

## Conclusion

There are 240 million children worldwide living with a disability, many of whom are at high risk for malnutrition. These vulnerable children continue to be routinely excluded from health services, nutrition programs, research, and even basic demographic and census data—all of which could improve their lives. Without tools to measure and count these children, they will continue to be underserved or excluded. MUAC is currently being used among children with disabilities and presents a potentially valuable part of anthropometric assessment for this population but there is a limited amount of interpretable or clear data on its use. Without validated measures to identify malnutrition and monitor the growth of these children, millions could have severe but avoidable consequences to their health and development. Future research should examine the use of MUAC both as part of multimodal nutrition assessments and as a tool for identifying children at high risk of malnutrition-associated adverse outcomes, especially when other anthropometric measurements may not be appropriate based on clinical sequelae.

## Supplemental Material

sj-docx-1-nah-10.1177_02601060231181607 - Supplemental material for Mid-upper arm circumference (MUAC) measurement usage among children with disabilities: A systematic review Supplemental material, sj-docx-1-nah-10.1177_02601060231181607 for Mid-upper arm circumference (MUAC) measurement usage among children with disabilities: A systematic review by Julia Hayes, Michael Quiring, Marko Kerac, Tracey Smythe, Cally J Tann, Nora Groce, Zerihun Gultie, Lydia Nyesigomwe and Emily DeLacey in Nutrition and Health

sj-docx-2-nah-10.1177_02601060231181607 - Supplemental material for Mid-upper arm circumference (MUAC) measurement usage among children with disabilities: A systematic review Supplemental material, sj-docx-2-nah-10.1177_02601060231181607 for Mid-upper arm circumference (MUAC) measurement usage among children with disabilities: A systematic review by Julia Hayes, Michael Quiring, Marko Kerac, Tracey Smythe, Cally J Tann, Nora Groce, Zerihun Gultie, Lydia Nyesigomwe and Emily DeLacey in Nutrition and Health
