# Supplementary material for: Mid-upper arm circumference (MUAC) measurement usage among children with disabilities: A systematic review
Source: Nutr Health. 2023 Jun 20;32(1):95–116. doi: 10.1177/02601060231181607 (PMC12982574; doi:10.1177/02601060231181607)
Supplement: sj-docx-1-nah-10.1177_02601060231181607 - Supplemental material for Mid-upper arm circumference (MUAC) measurement usage among children with disabilities: A systematic review [file sj-docx-1-nah-10.1177_02601060231181607.docx]

Appendix Materials:

Table A1. Inclusion/exclusion criteria for search study

Table A2. Full Search Strategy

Table A3. Quality appraisals

Table A4. Full Table 2- MUAC terminology, methods, references and comparison to other anthropometric indicators

Table A5. Excluded studies based on exclusion criteria

Supplementary Materials:

S1: PRISMA Checklist

**Appendix**

Table A1. Inclusion/exclusion criteria for search strategy.

| **Inclusion Criteria** | **Exclusion Criteria** |
| --- | --- |
| English | Non-English language |
| 6 months through 18 years old  Children with disabilities | Infants younger than 6 months  Adults older than 18 years old  No children with disabilities |
| All geographic locations | Intensive care units |
| Mid-upper arm circumference measurement | No mid-upper arm circumference measurement |
| Measures of nutritional status or health outcome | No measures of nutritional status |
| Published January 1990 through September 2021 | Published before 1990 |

Table A2. Full search strategy for systematic review developed with guidance from Banks et. al. [27].

| 1 | ((arm OR midarm OR mid-arm) AND circumference) OR MUAC |
| --- | --- |
| 2 | MH (Malnutrition OR Protein-Energy Malnutrition OR Nutritional Status OR Nutrition) OR malnutrition OR nutrition* status OR undernutrition OR nutrition* deficiency OR nutrition |
| 3 | MH (Nutrition Assessment) OR (nutrition* AND (assessment OR screen* OR evaluation OR measurement)) |
| 4 | 2 OR 3 |
| 5 | MH (Child OR Preschool OR Adolescents OR Infants) OR child* OR preschool* OR adolescen* OR infan* OR baby OR babies OR young people* OR young person* OR youth OR teen* |
| 6 | MH (Disabled persons OR Disabled children) OR disabilit* OR disabled OR handicap* |
| 7 | Physical impair* or physically impair* OR physical deficien* OR physically deficien* OR physical disab* OR physically disab* OR physical handicap* OR physically handicap* OR physically challeng* |
| 8 | MH (Cerebral palsy OR Arthritis spinal dysraphism OR muscular dystrophies OR Musculoskeletal Abnormalities OR Chronic Brain Injury OR Poliomyelitis OR Paraplegia OR Hemiplegia) OR Cerebral pals* OR Spina bifida OR Muscular dystroph* OR Arthriti* OR Osteogenesis imperfecta OR Musculoskeletal abnormalit* OR Musculo-skeletal abnormalit* OR Muscular abnormalit* OR Skeletal abnormalit* OR limb abnormalit* OR Amputation* OR Amputee OR Clubfoot OR Polio* OR Paraplegi* OR Paralys* OR Paralyz* OR Hemiplegi* |
| 9 | MH (Hearing loss) OR (Hearing loss* OR hearing impair* OR hearing deficien* OR hearing disable* OR hearing disabili* OR hearing handicap* OR acoustic loss* OR acoustic impair* OR acoustic deficien* OR acoustic disable* OR acoustic disabili* OR acoustic handicap* OR Deaf* or hearing loss) |
| 10 | MH (Blindness) OR (vision loss* OR vision impair* OR vision deficien* OR vision disable* OR vision disabili* OR vision handicap* OR visual loss* OR visual impair* OR visually impair* OR visual deficien* OR visually deficien* OR visual disable* OR visually disable* OR visual disabili* OR visually disabili* OR visual handicap* OR visually handicap* OR low vision OR reduced vision NOT double blind* NOT blinding NOT triple blind*) |
| 11 | MH (schizophrenia and disorders with psychotic features) OR (Mental disorder* OR Schizophreni* OR Psychosis OR psychoses OR Psychotic Disorder* OR Schizoaffective Disorder* OR Schizophreniform Disorder*) |
| 12 | (intellectual illness* OR intellectual impair* OR intellectual deficien* OR intellectual disable* OR intellectual disabili* OR intellectual handicap* OR intellectual retard* OR mental ill OR mentally ill OR mental illness* OR mental impair* OR mentally impair* OR mental deficien* OR mentally deficien* OR mental disable* OR mentally disable* OR mental disabili* OR mental handicap* OR mentally handicap* OR developmental impair* OR developmentally impair* OR developmental deficien* OR developmentally deficien* OR developmental disable* OR developmentally disable* OR developmental disabili* OR developmentally disabili* OR developmental handicap* OR developmentally handicap* OR developmental retard* OR developmentally retard* OR psychological ill OR psychologically ill OR psychological illness* OR psychological impair* OR psychologically impair* OR psychological deficien* OR psychologically deficien* OR psychological disable* OR psychologically disable* OR psychological disabili* OR psychological handicap* OR psychologically handicap*) |
| 13 | MH (Learning disorders OR communication disorders) OR (learning disorder* OR communication disorder* OR language disorder* OR speech disorder* OR speech disorder*) |
| 14 | MH (Pervasive Child Development Disorders) OR (autistic OR autism OR asperger* or dyslexi* OR Down’s Syndrome OR Down Syndrome OR Mongolism or Trisomy 21) |
| 15 | 6 OR 7 OR 8 OR 9 OR 10 OR 11 OR 12 OR 13 OR 14 |
| 16 | 1 AND 4 AND 5 AND 15 |

Table A3. Quality appraisals for cross sectional, cohort, case-control and randomized control trial studies using the JBI Critical Appraisal Tool [30].

**Table A3.1** Cross sectional studies

| **Author** | **Year** | **1. Were the criteria for inclusion in the sample clearly defined?** | **2. Were the study subjects and the setting described in detail?** | **3. Was the exposure measured in a valid and reliable way?** | **4. Were objective, standard criteria used for measurement of the condition?** | **5. Were confounding factors identified?** | **6. Were strategies to deal with confounding factors stated?** | **7. Were the outcomes measured in a valid and reliable way?** | **8. Was appropriate statistical analysis used?** | **Overall appraisal** **(include, exclude or seek further information)** |
| --- | --- | --- | --- | --- | --- | --- | --- | --- | --- | --- |
| A. Hussain | 1996 | Y | Y | Y | Y | N | N/A | Y | Y | Include |
| J. Tompsett | 1999 | Y | Y | Y | Y | N | N/A | Y | Y | Include |
| S. Zainah | 2001 | Y | Y | Y | Y | N | N/A | Y | Y | Include |
| K. Troughton | 2001 | Y | Y | Y | Y | N/A | N/A | Y | Y | Include |
| A. Dannhauser | 2007 | Y | Y | Y | Y | N/A | N/A | Y | Y | Include |
| HY. Tomoum | 2010 | Y | Y | Y | Y | N | N/A | Y | Y | Include |
| A. Al-Saqladi | 2010 | Y | Y | Y | Y | N/A | N/A | Y | Y | Include |
| R. Hamza | 2015 | Y | Y | Y | Y | N/A | N/A | Y | Y | Include |
| B. Silva | 2017 | Y | Y | Y | Y | N/A | N/A | Y | Y | Include |
| P. Saldanha | 2018 | Y | Y | Y | Y | N | N | Y | Y | Include |
| H. Tekin | 2018 | Y | Y | Y | Y | Y | Y | Y | Y | Include |
| K. Rose-Clarke | 2019 | Y | Y | Y | Y | N | N | Y | Y | Include |
| R. Ahmad | 2020 | Y | Y | Y | Y | Y | Y | Y | Y | Include |
| A. Kotby | 2020 | Y | Y | Y | Y | N | N | Y | Y | Include |
| J. Saleem | 2021 | Y | Y | Y | Unclear | Y | N | Y | Y | Include |
| M. Zenitani | 2021 | Y | Y | Unclear | Unclear | N | N | Y | Y | Exclude |
| I. Jahan | 2021 | Y | Y | Y | Y | Y | Y | Y | Y | Include |
| I. Jahan | 2021 | Y | Y | Y | Y | Y | Y | Y | Y | Include |
| H. Sahin | 2021 | Y | Y | Y | Y | Y | N | Y | Y | Include |
| D. Arony | 2018 |  |  |  |  |  |  |  |  | Exclude |
| M. Leonard | 2020 | Y | Y | Y | Y | Y | N | Y | Y | Include |
| DeLacey | 2021 | Y | Y | Y | Y | Y | Y | Y | Y | Include |

Table A3.2 Cohort Studies

| **Author** | **Year** | **1.Were the two groups similar and recruited from the same population?** | **2.Were the exposures measured similarly to assign people to both exposed and unexposed groups?** | **3.Was the exposure measured in a valid and reliable way?** | **4.Were confounding factors identified?** | **5.Were strategies to deal with confounding factors stated?** | **6.Were the groups/participants free of the outcome at the start of the study (or at the moment of exposure)?** | **7.Were the outcomes measured in a valid and reliable way?** | **8. Was the follow up time reported and sufficient to be long enough for outcomes to occur?** | **9.Was follow up complete, and if not, were the reasons for loss to follow up described and explored?** | **10.Were strategies to address incomplete follow up utilized?** | **11.Was appropriate statistical analysis used?** | **Overall appraisal** **(include, exclude or seek further information)** |
| --- | --- | --- | --- | --- | --- | --- | --- | --- | --- | --- | --- | --- | --- |
| DJ. Bartlett | 2010 | Y | Y | Y | Y | Y | Y | Y | Y | Y | Y | Y | Include |
| M. Kuperminc | 2010 | Y | Y | Y | Y | Y | Y | Y | Y | Y | Y | Y | Include |
| M. Samara | 2010 | Y | Y | Y | Y | Y | Y | Y | Y | Y | Y | Y | Include |
| J. Tumwine | 2015 | Y | Y | Y | Y | Y | Y | Y | Y | Y | Y | Y | Include |
| N. Lelijveld | 2016 | Y | Y | Y | Y | Y | Y | Y | Y | Y | Y | Y | Include |

Table A3.3 Case control studies

| **Author** | **Year** | **1.Were the groups comparable other than the presence of disease in cases or the absence of disease in controls?** | **2.Were cases and controls matched appropriately?** | **3.Were the same criteria used for identification of cases and controls?** | **4.Was exposure measured in a standard, valid and reliable way?** | **5.Was exposure measured in the same way for cases and controls?** | **6.Were confounding factors identified?** | **7.Were strategies to deal with confounding factors stated?** | **8.Were outcomes assessed in a standard, valid and reliable way for cases and controls?** | **9.Was the exposure period of interest long enough to be meaningful?** | **10.Was appropriate statistical analysis used?** | **Overall appraisal** **(include, exclude or seek further information)** |
| --- | --- | --- | --- | --- | --- | --- | --- | --- | --- | --- | --- | --- |
| M. Pai | 2001 | Y | Y | Y | Y | Y | N | N/A | Y | Y | Y | Include |
| A. Freeman | 2002 | Unclear | Unclear | Y | Y | Y | N | N/A | Y | Y | Y | Include |
| H. Kuper | 2015 | Y | Y | Y | Y | Y | Y | Y | Y | Y | Y | Include |
| K. Barnhill | 2017 | Y | Y | Y | Y | Y | Y | Y | Y | Y | Y | Include |
| C. Caminiti | 2018 | Y | Y | Y | Y | Y | N | Y | Y | Y | Y | Include |

Table A3.4 RCT

| **Author** | **Year** | **1.Was true randomization used for assignment of participants to treatment groups?** | **2.Was allocation to treatment groups concealed?** | **3.Were treatment groups similar at the baseline?** | **4.Were participants blind to treatment assignment?** | **5.Were those delivering treatment blind to treatment assignment?** | **6.Were outcomes assessors blind to treatment assignment?** | **7.Were treatment groups treated identically other than the intervention of interest?** | **8.Was follow up complete and if not, were differences between groups in terms of their follow up adequately described and analyzed?** | **9.Were participants analyzed in the groups to which they were randomized?** | **10.Were outcomes measured in the same way for treatment groups?** | **11.Were outcomes measured in a reliable way?** | **12.Was appropriate statistical analysis used?** | **13.Was the trial design appropriate, and any deviations from the standard RCT design (individual randomization, parallel groups) accounted for in the conduct and analysis of the trial?** | **Overall appraisal** **(include, exclude or seek further information)** |
| --- | --- | --- | --- | --- | --- | --- | --- | --- | --- | --- | --- | --- | --- | --- | --- |
| B. Zemel | 2002 | Y | Unclear | Y | Unclear | Unclear | Unclear | Y | Y | Y | Y | Y | Y | Y | Include |
| O. Soylu | 2008 | Y | Y | Y | Y | Y | Y | Y | Y | Y | Y | Y | Y | Y | Include |

Table A4. Full Table 2- MUAC terminology, methods, references and comparison to other anthropometric indicators.

| **Author, Year** | **MUAC Terminology** | **How MUAC was Measured** | **Reference for Measurements** | **MUAC Results** | **BMI** | **Height-for-age** | **Weight-for-age** | **Weight-for-height** | **Other Anthropometry** |
| --- | --- | --- | --- | --- | --- | --- | --- | --- | --- |
| African Region | | | | | | | | | |
| Dannhauser et al., 2007 | mid upper-arm circumference (MUAC) | Right side  Average of 3 measurements | National Centre for Health Statistics | MEAN (SD)  Martie du Plessis (13) - 19.2 SD 6.2  Pholoho (64) - 17.2 SD 4.9  Tswellang (67) - 17.0 SD 4.4  Percentage of children in percentile category MDP, Pho, Tsw (<5): 15.4, 3.2, 0 (5-<15): 15.4, 41.2, 56.7 (15 - <85): 53.8, 50.8, 38.8 (85 - <95): 7.7, 1.6, 1.5 (>/= 95): 7.7, 3.2, 3.0 |  | Reported as percentage of group within z-score deviation category  (< –3) MDP 1 (7.7) Pho 6 (9.3) Tsw 21 (31.3)  (–3 - < –2) MDP 1 (7.7) Pho 18 (28.1) Tsw 11 (16.4)  (–2 - < –1) MDP 5 (38.5) Pho 16 (25.0) Tsw 16 (23.9)  (–1 - < 1) MDP 4 (30.8) Pho 22 (34.4) Tsw 18 (26.9)  (> 1 - < 2) MDP 2 (15.4) Pho 1 (1.6) Tsw1 (1.5)  (>/=2) MDP 0 (0) Pho 1 (1.6) Tsw 0 (0) | Reported as percentage of group within z-score deviation category  MDP, Pho, Tsw (< –3): 7.7, 0, 10.5 (–3 - < –2): 30.7, 18.7, 19.4 (–2 - < –1): 7.7, 35.9, 29.9 (–1 - < 1): 23.1, 43.8, 38.8 (1 - < 2): 23.1, 0, 0 (>/= 2): 7.7, 1.6, 1.5 | Reported as percentage of group within z-score deviation category  MDP, Pho, Tsw (< –3): 0, 0, 4.5 (–3 - < –2): 0, 1.6, 1.5 (–2 - < –1): 7.7, 3.1, 9.0 (–1 - < 1): 7.7, 18.8, 19.4 (> 1 - < 2): 0, 3.1, 4.5 (>/= 2): 84.6, 73.4, 61.1 | Triceps skinfold (mm)  Martie du Plessis (13) - 15.6 SD 14.4  Pholoho (63) - 11.2 SD 5.1  Tswellang (67) - 9.3 SD 4.0  Percentage of children in percentile category (<5): 15.4, 0, 0 (5 - <15): 0, 14.3, 23.9 (15 - <85): 69.2, 80.9, 70.1 (85 - <95): 0, 3.2, 4.5 (>/= 95): 15.4, 1.6, 1.5   Others:  Upper-arm muscle area, upper-arm fat area |
| Kakooza-Mwesige et al., 2015 | mid-upper arm circumference | Tape measurer  Measured to the nearest 0.1 cm  Average of 2 measurements | WHO growth standards | Z-SCORE Mean (SD)  n=94 -0.38 (1.17) | Z-SCORE Mean (SD)  n=131 -0.92 (1.56) | Z-SCORE Mean (SD)  n=128 -1.57 (1.57) | Z-SCORE Mean (SD)  n=127 -1.57 (1.48) | Z-SCORE Mean (SD)  n=94 -0.84 (1.41) | Head circumference Z-SCORE Mean (SD)  n=94 -1.08 (2.00) |
| Kuper et al., 2015 | mid Upper Arm Circumference | Child tapes  Measured to the nearest 0.1 cm  Average of 3 measurements | WHO Child Growth Standards | MUAC (reported as the number of children (percentage) in range) Low MUAC for age: (z-score -2 or less) Children with disabilities: 39 (25%) Sibling Control: 17 (15%) Neighborhood Control: 17 (10%)   MUAC for height MEAN (SD)  n=155 -1.4 (1.3) | Reported as the number of children (percentage) in the range Low BMI for age (z-score -2 or less) Children with disabilities: 84 (37%) Sibling Control: 47 (26%) Neighborhood Control: 68 (24%)  MEAN (SD)  n=227 -1.6 (1.3) | Reported as the number of children (percentage) in the range Low height for age (z-score -2 or less) Children with disabilities: 77 (34%) Sibling Control: 42 (23%) Neighborhood Control: 58 (21%)  MEAN (SD)  n=225  -1.4 (1.8) | Reported as the number of children (percentage) in the range Low cutoff (z-score -2 or less) Children with disabilities: 158 (54%) Sibling Control: 63 (34%) Neighborhood Control: 86 (30%)   MEAN (SD)  n=294 -2.1 (1.6) | Reported as the number of children (percentage) in the range Low cutoff (z-score -2 or less) Children with disabilities: 39 (33%) Sibling Control: 26 (23%) Neighborhood Control: 31 (20%)   MEAN (SD)  n=120  -1.5 (1.4) |  |
| Lelijveld et al., 2016 | mid-upper arm circumference | Measured in mm | Guidelines by Lohman and colleagues and WHO | MEAN (SD)  Cases: 172 (20) Sibling controls: 183 (29.8) P value: 0.002 Community controls: 178 (22) P value: 0.001 | MEAN (SD)  Cases: -0.8 (0.9) Sibling controls: -0.8 (0.9) P value: 0.39 Community Control: -0.7 (0.9) P value: 0.31 | MEAN (SD)  Cases: -1.8 (1.2) Sibling controls: 1.5 (1.2) P value: 0.04 Community Control: -1.3 (1.1) P value: 0.001 | MEAN (SD)  Cases: -1.6 (0.9) Sibling controls: -1.4 (1.0) P value: 0.16 Community Control: -1.2 (0.9) P value: 0.06 |  | Standing height: MEAN (SD) Cases: 124.9 (9.0)  Controls:130.3 (16.8) P value: 0.004 Community Control: 127.4 (9.9) P value: 0.001  Head circumference: MEAN (SD) Cases: 51.1 (2.1) Sibling Controls: 52.1 P value:0.31 Community Control: 52.1 (1.9) P value: 0.12  Others: leg length, sitting height, calf circumference, R/height, Xc/height, phase angle, waist circumference, hip circumference, waist to hip ratio |
| Tompsett et al.,1999 | mid-upper arm circumference (MUAC) | Left arm  Sitting position  Measured to the nearest 0.1 cm | Standard methods (United Nations, 1986) | MEAN +/- S.D.  Disabled: 16.0 +/- 1.6 Siblings: 15.8 +/- 1.6 Neighbors: 15.6 +/- 1.3 |  | Reported as Z-score for group Disabled: -2.5+/-1.8 (66/112) Siblings: -1.8+/-1.8 (39/87) Neighbors: -1.3 +/- 1.6 (37/112) | Reported as Z-score for group Disabled: -1.6 +/-1.1 (42/112) Siblings: -1.1 +/1 1.3 (14/87) Neighbors: -1.0 +/- 1.1 (13/112) | Reported as Z-score for group Disabled: -0.0 +/- 1.9 (7/112) Siblings: 0.5 +/- 2.4 (2/87) Neighbors: 0.0 +/- 1.9 (1/112) |  |
| Region of the Americas | | | | | | | | | |
| Barnhill et al., 2017 | mid-arm circumference (MAC) | Standard steel precision calipers | CDC charts and the percentile rankings | No values. With regard to MAC measurements for children with and without ASD, no participants met the criteria for any category of undernutrition or risk of undernutrition. | BMI MEAN (SD) Cases: 16.15 (2.04) Controls: 16.32 (1.88)  BMI Z-Score Cases 0.16 (1.17) Controls: 0.25 (1.07) | MEAN (SD) Height (cm) Cases: 112.13 (12.31) Controls:117.52 (13.65) | MEAN (SD) Weight (kg) Cases: 20.55 (5.35) Controls:22.91 (5.94) |  | MAMC -  Cases: <5th percentile: 26 (30.6%) 5-10th: 15 (17.64%) 10-25th: 7 (8.2%) 25-50th: 19 (22.35%) 50-75th: 12 (14.11%) 75th-90th: 2 (2.35%) >90th: 4 (4.71%)  Controls: <5th percentile: 26 (30.6%) 5-10th: 15 (17.64%) 10-25th: 7 (8.2%) 25-50th: 19 (22.35%) 50-75th: 12 (14.11%) 75th-90th: 2 (2.35%) >90th: The results indicate no significant association between ASD status and MAMC percentile range as measured by Fisher’s exact test (P = 0.268). |
| Bartlett et al., 2010 | mid-arm circumference | Left side  Sitting down  Measured in cm  Obtained by 2 raters to determine acceptable variation | Standard methods | Correlations between potential determinant and drop in GMFM-66 scores  Mean mid-arm circumference: n=135 Correlation coefficient ( -0.02 -0.16, 0.12) p-value 0.81  Change in mid-arm circumference:  n=103 Correlation coefficient -0.23 (-0.38, -0.07) p-value 0.02 |  |  |  |  | Correlations between potential determinants and drop in GMFM-66 scores  Mean triceps skinfold: n=134 Correlation coefficient -0.07 (-0.21, 0.08) p-value 0.44  Change in triceps skinfold: n=101 Correlation coefficient-0.19 (-0.35, -0.03) p-value 0.06 |
| Caminiti et al., 2018 | arm circumference | Non-extensible tape measure  Mid-point between the olecranon process and the acromion  Measured in cm | WHO’s standards up to 60 months old Argentine references as of 61 months old | MEAN +/- SD (cm)  20.5 ± 5.7 | MEAN (SD)  BMI (kg/height^2^) 19 ± 4.8 BMI Z-score 0.88 (-5.3/4.4) | MEAN (SD)  Height (cm) 116 ± 29.3 Height Z-score -1.63 (-7.5/3.9) | MEAN (SD)  Weight (kg) 22 (6.3/79) Weight Z-score -0.2 (-4.4/4) |  | Waist (CM): 65.2 ± 12  Tricipital skinfold (mm): 10.5 ± 3.9  Others: Bicipital skinfold (mm), Subscapular skinfold (mm), Suprailiac skinfold (mm) |
| Kuperminc et al., 2010 | mid-upper arm circumference | Average of two measurements | Standardized measures | Z-SCORES (SD)  Cases: -0.8 (1.3) References: 0.5 (1.3)  Represented as R2 values to quantify amount of variation observed in DXA percentage body fat explained by each anthropometric measure.  (CP R2 =0.30; NHANES R2 =0.43) | Z-SCORES (SD)  Cases: -0.7 (2.0) References: 0.5 (1.1)  Represented as R2 values to quantify amount of variation observed in DXA percentage body fat explained by each anthropometric measure.  CP R2 =0.30; NHANES R2 =0.43 |  |  |  | Z-SCORES (SD)  Cases: -0.1 (1.1) References: 0.5 (1.2) |
| Saldanha et al., 2018 | arm circumference | Flexed the elbow at 90 degrees with the palm facing up  Calculated distance between anatomical landmarks and the central point is marked with a demographic pencil  Anthropometric tape fixed on marked point | WHO growth curves 2007 | MEAN +/- SD (cm)  22.09 ± 6.33 | MEAN +/- SD BMI (kg/m2)  18.43 ± 5.87 | MEAN +/- SD Height (m)  1.27 ± 0.27 | MEAN +/- SD Weight (Kg)  33.72 ± 26-21 |  | Triceps skinfold - MEAN +/- SD (mm) 16.44 ± 12.00 |
| Silva et al., 2017 | arm circumference | Inextensible tape  Three measurements obtained, closest two were averaged and reported | WHO: weight, estimated height, BMI Frisancho: brachial circumference, tricep skinfold, and arm circumference | Reported as number of children (percentage) in range  Under 91% percentile: n=25 (36.8) | Reported as number of children (percentage) in range  Less than or equal to -2.0 z-score: n=29, 42.6% | Reported as number of children (percentage) in range  Less than or equal to -2.0 z-score: n=26, 38.2% | Reported as number of children (percentage) in range  Less than or equal to -2.0 z-score: n=17, 27.0% |  | Triceps skinfold thickness Arm muscle circumference |
| Zemel et al., 2002 | arm circumference (AC) | Non-stretchable tape  Measured to the nearest 0.1 cm  Mean value of 3 measurements | Unspecified | MEAN +/- S.D. MUAC (cm)   Baseline -  Control: 17.0 ± 2.4 Zinc: 16.5 ± 1.2   12 months Control: 17.6 ± 3.1 Zinc: 17.5 ± 1.4  Z SCORES  Baseline -  Control: 1.12 ± 1.19 Zinc: 1.19 ± 0.70   12 months -  Control: 1.18 ± 1.16 Zinc: 0.93 ± 0.77 | MEAN +/- S.D.  Baseline -  Control: 14.9 ± 2.2 Zinc: 14.8 ± 0.8   12 months Control: 15.0 ± 2.3 Zinc: 15.0 ± 1.1 | Reported as z-score for group at baseline and 12 months  Baseline  Control: 0.28 ± 1.04  Zinc: 0.42 ± 1.02   12 months Control: 0.23 ± 1.14  Zinc: 0.35 ± 1.03 | Reported as z-score for group at baseline and 12 months  Baseline: Control: 0.74 ± 1.22  Zinc: 0.71 ± 0.86   12 months Control: 0.77 ± 1.21  Zinc: 0.68 ± 1.00 |  | BMI-for-age (z-score)  Baseline: Control : 0.91 ± 1.31 Zinc: 0.57 ± 0.62  12 months Control: 1.03 ± 4.5 Zinc: 0.65 ± 0.76  Triceps skinfold thickness (mm)  Baseline: Control: 6.9 ± 3.1 Zinc: 7.8 ± 3.1  12 months Control: 6.6 ± 5.0 Zinc: 7.2 ± 2.4  Others: Weight, arm muscle area, arm fat area, triceps skinfold thickness z-score, UAMA z-score, UAFA z-score |
| South-East Asia Region | | | | | | | | | |
| Freeman et al., 2002 | mid-upper-arm circumference (MUAC) | Unspecified | UN Guidelines (United Nations, 1986) | MEAN +/- S.D. MUAC (cm)   Disabled: Male - 14.72 (1.27), Female - 15.24 (1.50)  Non-Disabled: Male - 15.17 (1.38), Female - 14.95 (1.17) | MEAN +/- S.D BMI (kg/m2)d  Disabled: Male: 14.36 (1.16), Female: 14.43 (1.47)  Non-disabled: Male: 15.09 (1.56), Female: 14.02 (1.18) | Reported as z-score for disabled male and female vs non-disable male and female  Disabled: Male: -2.64 (1.80), Female: -1.73 (1.80)   Non-disabled: Male: -2.69 (1.84), Female: -1.53 (1.77) | Reported as z-score for disabled male and female vs non-disable male and female  Disabled: Male: -2.43 (1.11), Female: -1.80 (1.33)  Non-disabled: Male: -2.16 (1.09), Female: -1.92 (1.05) | Reported as z-score for disabled male and female vs non-disable male and female  Disabled: Male: -1.23 (0.75), Female: -0.98 (0.91)  Non-disabled: Male: -2.69 (0.84), Female: -1.22 (0.81) | Subscapular skinfold (cm) Disabled: Male: 6.70 (1.96), Female: 8.14 (1.91)  Non-disabled: Male: 6.84 (1.62), Female: 7.54 (1.49)  Triceps skinfold (cm) Disabled: Male: 7.84 (2.25), Female: 10.04 (1.91)  Non-disabled: Male: 9.93 (1.83), Female: 10.03 (1.41) |
| Hussain et al., 1996 | mid-upper arm circumference (MUAC) | Left upper arm  Measured to the nearest cm  Oil-cloth tailor's tape | National Center for Health Statistics  US NHANES 1 | PERCENTILE  Normal: >85% Cases: 8, Controls 24 OR 1.0 Moderate: 80-84% Cases: 20, Controls: 22 OR 2.7 (0.9-8.4) Severe: <80% Cases: 43, Controls: 24 OR 5.4 (1.9-15.5) |  | Reported as number of children in range For those with a confirmed dx: Normal: > -2 H/A Cases 31, Controls 37 OR Moderately stunted: -2 to -2.99 H/A Cases 16 Controls 17, OR 1.1 (0.4-2.8) Severely stunted: </= -3 H/A Cases 24, Controls 16 OR 1.8 (0. 7-4.3) | Reported as number of children in range Normal: > -2 W/A Cases 23, Controls 37 OR 1.0 Moderately wasted: -2 to -2.99 W/A Cases 28, Controls 20, OR 1.6 (0.7-3.9) Severely wasted: </= -3 W/A Cases 20, Controls 13, OR 2.5 (1.0-6.5) | Reported as number of children in range Normal: > -2 W/H Cases 52, Controls 54, OR 1.0 Moderately wasted: -2 to -2.99 W.H Cases 18, Controls 16, OR 1.2 (0.5-2.7) Severely wasted: </= -3 W/H Cases 1, Controls 0, OR - |  |
| Jahan et al., 2019 | mid-upper arm circumference (MUAC) | Measured in cm  MUAC tapes | WHO cut-offs | Reported as number (percentage) within range  Normal: 9 (11.3%)  Undernutrition: 8 (10.0%)  Severe undernutrition: 63 (78.8% | Reported as number (percentage) within range  Normal: 81 (64.8%)  Undernutrition: 19 (15.2%)  Severe undernutrition: 25 (20.0%) | Reported as number (percentage) within range  Normal: 9 (7%)  Undernutrition: 9 (7%)  Severe undernutrition: 110 (85.9%) | Reported as number (percentage) within range  Normal: 9 (11.3%)  Undernutrition: 8 (10.0%)  Severe undernutrition:63 (78.8%) | Reported as number (percentage) within range  Normal: 24 (75%)  Undernutrition: 1 (3.1%)  Severe undernutrition: 7 (21.9%) |  |
| Jahan et al., 2021 | mid-upper-arm-circumference | Measured in cm  MUAC tapes | WHO protocol | MEAN (SD): −0.9 (1.4)  Reported as number (percentage) within range Overnutrition: (z score: >+2 SD): 0 Normal: (z score: −2 SD to +2 SD): 21 (75) Moderate undernutrition: (z score: >−3 SD to <−2.0 SD): 3 (10.7) Severe undernutrition: (z score: ≤−3.0 SD): 4 (14.3) | MEAN (SD): −0.5 (4.1)  Reported as number (percentage) within range Overnutrition: (z score: >+2 SD): 13 (7.9) Normal: (z score: −2 SD to +2 SD): 103 (62.8) Moderate undernutrition: (z score: >−3 SD to <−2.0 SD): 19 (11.6) Severe undernutrition: (z score: ≤−3.0 SD): 29 (17.7) | MEAN (SD): −2.9 (2.6)  Reported as number (percentage) within range Overnutrition: (z score: >+2 SD): 6 (3.5) Normal: (z score: −2 SD to +2 SD): 55 (32.4) Moderate undernutrition: (z score: >−3 SD to <−2.0 SD): 30 (17.6) Severe undernutrition: (z score: ≤−3.0 SD): 79 (46.5) | MEAN (SD): −2.2 (1.9)  Reported as number (percentage) within range Overnutrition: (z score: >+2 SD): 4 (4.6)  Normal: (z score: −2 SD to +2 SD): 38 (43.7) Moderate undernutrition: (z score: >−3 SD to <−2.0 SD): 11 (12.6) Severe undernutrition: (z score: ≤−3.0 SD): 34 (39.1) | MEAN (SD): −0.5 (1.6)   Reported as number (percentage) within range Overnutrition: (z score: >+2 SD): 1 (3.8) Normal: (z score: −2 SD to +2 SD): 21 (80.8) Moderate undernutrition: (z score: >−3 SD to <−2.0 SD): 2 (7.7) Severe undernutrition: (z score: ≤−3.0 SD): 2 (7.7) |  |
| Pai et al., 2001 | mid-upper arm circumference (MUAC) | Measured to the nearest 0.1 cm | National Center for Health Statistics reference data | MEAN +/- S.D. MUAC (cm)   Disabled: 12.8(1.6)  Siblings: 13.2(1.4)   Neighbor Control: 13.0(1.3) |  | Reported as z-score for group   Disabled: 3.47(1.87)a –  Siblings: 2.50(1.66)b –  Neighbor Control: 2.78(1.74)b | WAZ (Z score) –Disabled: 2.78(1.25)   Sibling: 2.53(1.10) –  Neighbor Control: 2.37(0.95) | WHZ (Z score) Disabled–1.20(1.26)   Siblings: 1.46(1.30)  Neighbor Control: 1.05(0.84) |  |
| Rose-Clarke et al., 2019 Does not present data for disability only, all inclusive. | mid upper arm circumference (MUAC) | Standard adult tape (UNICEF)  Average of 2 measurements | WHO Reference 2007 | Reported as number of children (percentage) in range 10-14 years MUAC <160 mm 64 (3.6)  MUAC (SD)  10-19 years Mean  21.4 (2.8) | Reported as number of children (percentage) in range 15-19 years BMI <18.5 609 (40.8)  BMI <-2 SD 10-19 years 350 (10.7) | Reported as number of children (percentage) in range 15-19 years: 1488 (44.8) |  |  | Overweight BMI >1 SD Reported as number of children (percentage) in range 50 (1.5) |
| European Region | | | | | | | | | |
| Leonard et al., 2020 | mid-upper arm circumference (MUAC) | Unspecified | CDC charts for MUAC WHO charts for weight, height and BMI | In total, 35 of 82 patients whose MUAC was measured had a MUAC of < p10. |  |  |  |  | Specific anthropometrics not provided, only number of malnourished as determined by measurements.  In all, 55 children had acute malnutrition (28 moderate, 25 severe, two unclassified), 47 children had chronic malnutrition (29 moderate, 18 severe) (Fig. 1); 13 were malnourished both acutely and chronically (mixed malnutrition). A total of 22 children were obese; one of these also had chronic malnutrition. A normal nutritional status was found for 140 children. A nutritional diagnosis could not be attributed to 10 children: 7 with no anthropometric data recorded; 2 with a very low height. |
| Sahin and Nogay, 2021 | mid-upper arm circumference (MUAC) | Appropriate methods (WHO Technical Report Series 1995) | WHO – Child Growth Standards 2019, WHO Growth Reference Data 2007 | Reported as number (percentage) within range  Severe thinness: 19 (21.3%) Thinness: 18 (14.8%) Normal: 63 (51.6%) Overweight: 9 (7.4%) Obesity: 6 (4.9%) | Reported as number (percentage) within range  Severe thinness: 3 (2.5%) Thinness: 9 (7.4%) Normal: 71 (58.2%) Overweight: 15 (12.3%) Obesity: 24 (19.8%) | Reported as number (percentage) within range  Stunted: 24 (19.7%) Short: 32 (26.2%) Normal: 57 (46.7%) Tall: 4 (3.3%) Very Tall: 5 (4.1%) | Reported as number (percentage) within range  Severe thinness: 12 (9.8%) Thinness: 21 (17.2%) Normal: 30 (24.6%) Overweight: 29 (23.8%) Obesity: 30 (24.6%) |  |  |
| Samara et al., 2010 | mid-arm circumference | Average of 2 measurements  LASSO-O tape | Not stated | MEAN DIFFERENCE  Cases: 1.2 cm 95% CI 0.7-1.7cm; p<0.001 | MEAN DIFFERENCE  Cases: 1.2; 95% CI 0.8–1.7; p<0.001 | Height: (mean difference 3.1cm; 95% CI 1.6–4.6cm; p<0.001 | Weight: (mean difference 2.6kg; 95% CI 1.6–3.7kg; p<0.001 |  | Head circumference:  mean difference 1.4cm 95% CI 1.03–1.8cm; p<0.001 |
| Soylu et al., 2008 | midarm circumference | Left arm hanging down  Non-stretching tape  Midway between the olecranon and the acromion | Criteria of Waterlow | MEAN +/- S.D.  Midarm circumference (cm) 14.4 +/- 2.1  Midarm circumference (cm) Before Therapy 14.5 +/-2.2  After Therapy  15.2 +/-2.2 | Reported as mean +/- SD  Before: 13.6 +/- 2.1   After: 14.4 +/- 2.0 | Reported as percentage +/- SD  Before 89.4 +/- 8.8   After: 89.9 +/- 13.4 | Reported as percentage +/- SD  Before: 64.9 +/- 15.1   After: 66.2 +/- 19.2   Weight z-score Mean +/- SD: 2.1 +/- 0.9 Before therapy: 2.1 +/- 0.9 After therapy: 1.8 +/- 0.9 | Reported as percentage +/- SD  Before: 84.1 +/- 13.9   After: 88.7 +/- 13.4 | Head circumference (cm)  Mean +/- SD: 46.2 +/- 3.0 Before therapy: 46.3 +/- 3.1 After therapy: 46.0 +/- 3.4  Triceps skinfold thickness (mm) Mean +/- SD 9.6 +/- 3.4 Before therapy: 9.8 +/- 3.5 After therapy: 10.4 +/- 4.1   Others: weight, height, height s-score, |
| Tekin et al., 2018 | mid-upper arm circumference (MUAC) | Left upper arm flexed slightly at elbow  Half distance between the acromion and the olecranon  Plastic measuring tape | WHO growth standards | MEAN (SD) MUAC (cm)  Cases: Baseline: 15.8 (2.7)  6 months: 16.4 (2.9)  Controls: 18.9 (3.4) |  | MEAN (SD) and Percentage  Cases: Baseline: -1.0 (1.77) 6 months: -   Controls: 0.07 (1.23) | MEAN (SD)  Malnourished: Baseline: -2.38 (2.53)  6 months: -1.04 (0.35)  Non-malnourished: 0.16 (1.76) | MEAN (SD)  Cases: Baseline: 79.16 (9.23) 6 months: 81.42 (8.17)  Controls: 106.4 (16.71) | Triceps Skinfold Thickness:  MEAN (SD)  Cases: Baseline: 7.6 (3.1) 6 months: 7.7 (2.9)  Controls: 10.4 (3.2) |
| Troughton and Hill, 2001 | mid-arm circumference | Harpenden plastic tape  Average of at least 2 measurements | United States Health and Nutritional Survey 1 of 1971 to 1974 | PERCENTILE Mid-arm circumference <5th centile in 27 participants (30%). Overall 46% of participants (41 of 90) fulfilled the criteria for undernutrition. |  |  | Weight: 24 (27%) were <2nd centile |  | Subscapular skinfold: 5 (6%) were <3rd centile  Triceps skinfold: 17 (19%) were < 3rd centile |
| Eastern Mediterranean Region | | | | | | | | | |
| Al-Saqladi et al., 2010 | mid-upper arm circumference (MUAC) | Measured to the nearest 0.1 cm  Mid-point between the acromion and olecranon process  Left arm hanging loosely  Non-extensible fiberglass tape | WHO reference values (WHO Multicentre Growth Reference Study Group, 2006–2007) | Z-SCORES (SD)  All: -2.23 (1.02) Male: -2.11 (1.01) Female: -2.29 (1.03) Ages 6-11 months: -2.58 (1.42) 12-23 months: -1.67 (0.79) 24-35 months: -2.12 (1.07) 36-47 months: -2.39 (0.54) 48-60 months: -2.34 (1.01) | Z-SCORES (SD)  All: -1.21 (1.42) Male: -1.08 (1.53) Female: -1.29 (1.39) Ages 6-11 months: -2.04 (1.62) 12-23 months: -0.04 (1.68) 24-35 months: -0.83 (1.67) 36-47 months: -1.46 (0.99) 48-60 months: -1.61 (0.79) | Z-SCORES (SD)  All: -2.16 (1.23) Male: -2.79 (1.24) Female: -1.84 (1.12) Ages 6-11 months: 0.96 (1.13) 12-23 months: -2.60 (1.56) 24-35 months: -2.50 (1.33) 36-47 months: -2.21 (1.21) 48-60 months: -2.08 (0.90) | Z-SCORES (SD)  All: -2.17 ( 1.03) Male: -2.40 (0.96) Female: -2.05 (1.07) Ages 6-11 months: -1.83 (1.17) 12-23 months: -1.66 (1.03) 24-35 months: -2.16 (1.27) 36-47 months: -2.44 (0.67) 48-60 months: -2.41 (0.88) | Z-SCORES (SD)  All: -1.38 (1.29) Male: -1.31 (1.24) Female: -1.42 (1.33) Ages 6-11 months: -1.58 (1.48) 12-23 months: -0.53 (1.47) 24-35 months: -1.17 (1.59) 36-47 months: -1.68 (0.85) 48-60 months: -1.73 (0.85) |  |
| Hamza et al., 2015 | mid upper arm circumference | Average of 3 measurements  Measured to the nearest 0.1 cm  Left arm at 90 degrees across the body  Conventional non-stretchable tape  Midpoint between inferior border of the acromion and the tip of the olecranon | Norms of Frisancho | MUAC Percentile MEAN +/- SD  56±7.53 (3–97) | BMI SDS  MEAN +/- SD  –0.98±0.23 (2.28 to –3.7) | Height SDS  MEAN +/- SD  –3.52±0.55 (–1.01 to –6.22) | Weight SDS  MEAN +/- SD  –0.53±0.25 (2.29 to –3.50) |  | Head circumference  1.10±0.52 (–0.49 to 2.77)  Triceps skinfold thickness (TSFT) percentile   65±1.25 (3–98)  Subscapular skinfold thickness (SSFT) percentile 68±2.11 (3–98)  Others: RAS, SH |
| Kotby et al., 2020 | left mid-upper arm circumflex | Left arm  Upright with arm down in fully relaxed position  Tape measure perpendicular to the long axis of the arm  No punching or gaping of the tape | WHO global database | MEAN (SD) 13.65±2.46cm Range: 9-22cm  Cases: 13.79±2.79 Range: 9-22  Controls: 15.41±2.29 Range: 10.5-20 |  | Reported as number (percentage) within range  <-2: 36 (45.0%) -2 to 2 31 (38.8%) >2: 13 (16.3%) | Reported as number (percentage) within   <-2: 19 (23.8%) -2 to 2: 61 (76.3%) | Reported as number (percentage) within range  <-2: 9 (11.3%) -2 to 2: 55 (68.8%) >2: 16 (20.0%) |  |
| Saleem et al., 2021 | mid-upper arm circumference (MUAC) | Unspecified | WHO Child Growth Standards | MEAN (SD)  Cases: 9.97cm ±0.98cm  Controls: 14.00cm ±1.19cm |  | Cases:  Mean 66.82kg±9.58cm Mean HAZ: -3.94±1.41  Controls: Mean: 80.60kg±12.85cm Mean HAZ: -1.04±5.13 | Cases:  Mean: 5.39±1.69kg Mean WAZ: -4.64±1.07  Controls: Mean: 11.21±2.71kg -0.58±2.79 | Cases: Mean: 66.82±9.58cm Mean WHZ: -4.07±1.25  Controls: Mean: 80.60±12.85cm Mean WHZ: 0.40±1.27 |  |
| Tomoum et al., 2010 | mid-upper arm circumference (MAC) | Non-stretchable stainless-steel tape  Mean value of 3 measurements | Anthropometry Procedures Manual, National Center for Health Statistics, Centers for Disease Control and Prevention | No significant difference in MAC between patient and control groups. |  |  |  |  | Weight:  Males (cases): 14.3% < 10th percentile, 38.1% <50th percentile Females (cases): 15.8% < 10th percentile, 47.4% below 50th percentile  Cases (% of median[IQ Range]: 80.75[19.13] Controls (% of median[IQ Range]: 92.7[13.35]  Height: Male (cases): 4.8% < 10th percentile, 47.7% below 50th percentile Female (cases): 5.3% < 10th percentile, 78.9% below 50th percentile  Cases (% of median[IQ Range]: 91.15[9.35] Controls (% of median[IQ Range]: 98.05[9.38]  Head Circumference: Cases: 45.94±3.2 cm Controls: 50.03±1.17 cm  Waist Circumference: Cases (mean±SD): 46.72±5.09 cm Controls (mean±SD): 48.53±3.44 cm  Tricep Skinfold Thickness Cases (mean±SD): 8.31±2.60 cm Controls (mean±SD): 9.23±1.93 cm |
| Western Pacific Region | | | | | | | | | |
| Ahmad et al., 2020 | mid-upper-arm circumference (MUAC) | Measured in cm  Midpoint of the long axis of the upper arm  Wrapped around without compression of soft tissue | WHO | MEAN (SD) 18.7cm (5.37cm)  Reported as number of children (percentage) in range Normal: 52 (55.9%) Moderate Acute Malnutrition: 20 (21.5%) Severe Acute Malnutrition: 21 (22.6%) | MEAN (SD) -2.5 (3.14)  Reported as number of children (percentage) in range Overweight: 5 (5.8%) At risk of overweight: 12 (14.0%) Normal: 22 (25.6%) Thinness: 7 (8.1%) Severe Thinness: 40 (46.5%) | MEAN (SD)  −4.6 (1.92)  Reported as number of children (percentage) in range Normal: 2 (2.3%) Stunted: 14 (16.3%) Severe Stunted: 70 (81.4%) |  |  |  |
| Zainah et al., 2001 | mid-arm circumference | Left upper arm  Non-stretch measuring tape | Standardized methods | PERCENTILE and MEAN (SD)  <5 percentile -  CP - 52 Controls - 22 P-value <0.001  Mean (SD), difference between means 2.5 (– 3.50 to –1.43) CP - 18.8 (3.67) Controls - 17.61 (3.09) P Value 0.001 |  |  |  |  | Upper-arm length (cm) <5th percentile -  CP: 54 (53.5) Controls: 28 (27.7) P-value <0.001 Mean (SD) CP: 18.8 (3.67) Controls: 20.0 (3.61) P-value (<0.001) Difference between means: – 1.1 (– 1.65 to –0.59);  Tricep skinfold thickness (mm) <5th percentile -  CP: 40 (39.6) Controls: 7 (6.9) P-value <0.001 Mean (SD) CP: 7.1 (3.57) Controls: 9.5 (3.66) P-value (<0.001)  Weight (kg) <5th percentile -  CP: 79 (78.2) Controls: 15 (14.8) P-value <0.001 Mean (SD) CP: 15.1 (6.14) Controls: 21.1 (9.13) P-value (<0.001) |
| Multi-Region | | | | | | | | | |
| DeLacey et al., 2021 | Mid-upper arm circumference-for-age  Mid-upper arm circumference-for-age z-score (ACAZ) | For children 6 months - 5 years | WHO – Child Growth Standards 2019 | All children: Mid upper arm circumference-for-age z-score (ACAZ) (6 months to 5 years), n=426  -0.33±1.20  Children with disabilities:  6-12 months (n=9): -0.35 ± 1.58,  12-24 months (n=11): -0.70 ± 1.74 24-59 months (n=34): -0.73 ± 1.18  Children without disabilities:  6-12 months (n=60): -0.20 ± 1.19 12-24 months (n=88): -0.16 ± 1.21 24-59 months (n=223): -0.37 ± 1.14 | All children: BMI z-score (0–18 years) (n=2733) −0.62±1.45  Children with disabilities: 0-6 months (n=210): -1.79 ± 1.51  6-12 months (n=46): -1.63 ± 1.74 12-24 months (n=55): -1.04 ± 1.34 24-59 months (n=129): -0.92 ± 1.62 5-18 years (n=235): -0.56 ± 1.69  Children without disabilities: 0-6 months (n=643): -0.84 ± 1.39 6-12 months (n=102): -0.56 ± 1.38 12-24 months (n=137): 0.21 ± 1.25 24-59 months (n=287): -0.05 ± 1.22 5-18 years (n=888): -0.41 ± 1.23 | All children: Height-for-age z-score (0–18 years) (n=1686) −1.74±1.67  Children with disabilities: 0-6 months (n=192): -2.68 ± 1.73 6-12 months (n=45): -2.34 ± 1.85 12-24 months (n=54): -2.18 ± 1.31 24-59 months (n=132): -2.43 ± 1. 62 5-18 years (n=231): -1.98 ± 1.47   Children without disabilities: 0-6 months (n=713): -1.54 ± 1.73  6-12 months (n=105): -1.04 ± 1.62 12-24 months (n=142): -1.38 ± 1.46 24-59 months (n=303): -1.60 ± 1.29 5-18 years (n=895): -1.23 ± 1.24 | All children: Weight-for-age z-score (0–10 years) (n=2308) −1.48±1.54  Children with disabilities: 0-6 months (n=192): -2.68 ± 1.73 6-12 months (n=45): -2.34 ± 1.85 12-24 months (n=54): -2.18 ± 1.31 24-59 months (n=132): -2.43 ± 1. 62 5-18 years (n=231): -1.98 ± 1.47   Children without disabilities: 0-6 months (n=727): -1.48 ± 1.46 6-12 months (n=108): -1.04 ± 1.27 12-24 months (n=142): -0.69 ± 1.26 24-59 months (n=307): -1.09 ± 1.17 5-18 years (n=469): -0.99 ± 1.36 | All children: Weight-for-height z-score (0–5 years) (n=1678) −0.42±1.49  Children with disabilities: 0-6 months (n=189): -0.66 ± 1.61  6-12 months (n=46): -1.35 ± 1.72  12-24 months (n=55): -1.32 ± 1.33  24-59 months (n=130): -1.26 ± 1.58    Children without disabilities: 0-6 months (n=707): -0.20 ± 1.51 6-12 months (n=105): -0.48 ± 1.34 12-24 months (n=142): -0.03 ± 1.24 24-59 months (n=303): -0.25 ± 1.19 | All children: Head circumference-for-age z-score (0–5 years) (n=1095) −1.26±1.37  Children with disabilities 0-6 months (n=102): -2.36 ± 1.22 6-12 months (n=18): -2.09 ± 1.24 12-24 months (n=13): -0.82 ± 1.22 24-59 months (n=44): -1.18 ± 1.43  Children without disabilities: 0-6 months (n=483): -1.41 ± 1.41 6-12 months (n=77): -0.75 ± 1.33 12-24 months (n=111): -0.65 ± 1.12 24-59 months (n=247): -0.92 ± 1.23 |

Table A5. Excluded studies based on exclusion criteria.

| **#** | **Author, Year** | **Reason for Exclusion** |
| --- | --- | --- |
| 1 | Melunovic, 2017 | MUAC measurement not directly used as an anthropometric measurement for nutritional evaluation |
| 2 | Huysentruyt, 2018 | MUAC measurement not directly used as an anthropometric measurement for nutritional evaluation |
| 3 | Hasegawa, 2020 | MUAC measurement not directly used as an anthropometric measurement for nutritional evaluation |
| 4 | Gagil, 2001 | MUAC measurement not directly used as an anthropometric measurement for nutritional evaluation |
| 5 | Mortensen, 1990 | MUAC measurement not directly used as an anthropometric measurement for nutritional evaluation |
| 6 | Thommessen, 1991 | MUAC measurement not directly used as an anthropometric measurement for nutritional evaluation |
| 7 | Henderson, 1992 | MUAC measurement not directly used as an anthropometric measurement for nutritional evaluation |
| 8 | Strano, 1995 | MUAC measurement not directly used as an anthropometric measurement for nutritional evaluation |
| 9 | Samson-Fang, 2000 | MUAC measurement not directly used as an anthropometric measurement for nutritional evaluation |
| 10 | Lofthouse, 2002 | MUAC measurement not directly used as an anthropometric measurement for nutritional evaluation |
| 11 | Tuzun, 2013 | MUAC measurement not directly used as an anthropometric measurement for nutritional evaluation |
| 12 | Ponte, 2013 | MUAC measurement not directly used as an anthropometric measurement for nutritional evaluation |
| 13 | Nogay, 2013 | Non standardized |
| 14 | Pancheva, 2019 | Non standardized |
| 15 | Craig, 2006 | Includes subjects outside age range |
| 16 | Sanchez-Lastres, 2003 | Includes subjects outside age range |
| 17 | Schmitz, 2018 | Includes subjects outside age range |
| 18 | Zambrano, 2014 | Includes subjects outside age range |
| 19 | Teixeira, 2014 | Includes subjects outside age range |
| 20 | Caselli, 2017 | Includes subjects outside age range |
| 21 | Saleem, 2021 | Disability not significantly represented |
| 22 | Arony, 2018 | Excluded during critical appraisal |
| 23 | Zenitani, 2021 | Excluded during critical appraisal |
